# ICF Personal Factors Strengthen Commitment to Person-Centered Rehabilitation – A Scoping Review

**DOI:** 10.3389/fresc.2021.709682

**Published:** 2021-08-16

**Authors:** Maarit Karhula, Sari Saukkonen, Essi Xiong, Anu Kinnunen, Tuija Heiskanen, Heidi Anttila

**Affiliations:** ^1^Sustainable Well-being, Research and Development Department, South-Eastern Finland University of Applied Sciences, Mikkeli, Finland; ^2^Kela Research, Social Insurance Institution of Finland, Helsinki, Finland; ^3^Social Services and Health Care, Oulu University of Applied Sciences, Oulu, Finland; ^4^Social Services and Health Care, Savonia University of Applied Sciences, Kuopio, Finland; ^5^Public Health and Welfare Department, Knowledge Management and Co-Creation Unit, Functioning and Service Needs Team, Finnish Institute for Health and Welfare, Helsinki, Finland

**Keywords:** international classification of functioning disability and health, client-centeredness, person-centeredness, personal factors, rehabilitation, scoping review

## Abstract

**Background:** The International Classification of Functioning, Disability and Health (ICF) classification is a biopsychosocial frame of reference that contributes to a holistic understanding of the functioning of a client and the factors involved. Personal factors (PFs) are not currently classified in the ICF due to large societal and cultural diversity and lack of clarity in the scope of such factors.

**Aims:** To ascertain which factors in the ICF classification have been defined as PFs in different studies and what conclusions have been drawn on their role in the ICF classification.

**Methods:** The study was a scoping review. A systematic search for articles published in 2010–2020 was performed on the Cinahl, Pubmed, ScienceDirect, and Sport Discus databases. The PFs specified in the articles were classified according to the seven categories proposed by Geyh et al. socio-demographic factors; position in the immediate social and physical context; personal history and biography; feelings; thoughts and beliefs; motives; and general patterns of experience and behavior.

**Results:** The search yielded 1,988 studies, of which 226 met the inclusion criteria. The studies had addressed a wide variety of PFs that were linked to all seven categories defined by Geyh et al. Some studies had also defined PFs that were linkable to other components of the ICF or that did not describe functioning. Approximately 22% (51) of the studies discussed the role of PFs in rehabilitation.

**Conclusions:** The range of PFs in the ICF classification addressed in the reviewed studies is wide. PFs play an important role in rehabilitation. However, according to the reviewed studies, a more precise coding of PFs is not yet warranted.

## Introduction

Over the past 20 years, the International Classification of Functioning, Disability, and Health (ICF) has become a generally accepted biopsychosocial framework for rehabilitation (1). Through the provision of uniform concepts and a commonly shared frame of reference, the ICF classification has changed the practices and the statistics used to assess functioning and disability (2). However, the utilization of the ICF still needs to be further developed in the Nordic countries (3). In the ongoing rehabilitation reform led by the Finnish Ministry of Social Affairs and Health, the ICF classification is seen as a framework for establishing uniform practices in the assessment and documentation of functioning (4).

Enabling sufficient functioning is a complex process, as it comprises multiple interacting components that must be tailored to individual needs and situations (5). The Nordic countries appear to have a common conceptual understanding of client-centered practice (6), which is supported by applying the bio-psycho-social framework of the ICF in the complex processes of rehabilitation (7).

The ICF contains a broad range of categories for describing body functions and structures and activities and participation. In addition, environmental factors, which is one component of the contextual factors, can be defined as a barrier or a facilitator for functioning. However, the other component of contextual factors, personal factors (PFs), which are defined as the background information about the life and lifestyle of an individual, have not been classified (1). PFs include the resources, means of coping, education, and behavioral patterns of an individual. Identifying these functioning-related factors helps to understand how one's clients are, how they think, how they evaluate and understand their own situation, what they hope for, and how they cope in their daily lives. PFs and their interpretation influence the choice of rehabilitation services and measures, as well as other forms of support (8). Hence, the key question is how to identify and take into account the diverse PFs that affect the functioning of an individual in the same way as other factors included in the ICF classification.

It has been suggested that full utilization of the ICF classification is hindered by the fact that PFs are not categorized in the same way as their other components (9). Given the absence of a formal categorization of PFs in the ICF, studies have used various other categorizations. For example, in their review, Muller & Geyh (10) compared the background and content of eight different classifications. These classifications included, in varying degrees, the following 12 areas: socio-demographic factors, behavioral and lifestyle factors, cognitive psychological factors, social relationships, experiences and biography, coping, emotional factors, satisfaction, other health conditions, biological/physiological factors, personality, and motives/motivation. On the other hand, the use of a more precise classification of PFs has also been criticized. Leonardi et al. (11) suggested that PFs such as gender, age, or education may have implications for the disability of a person and are therefore important in understanding functioning. However, they did not favor a more precise classification of PFs, as this could lead to “blaming” clients for their functional limitations. Simeonsson et al. (12) proposed that before constructing a taxonomy of codes for PFs, one should critically assess the need for PFs as a separate component in the ICF classification. These conflicting views suggest that there is a need to systematically examine how PFs are defined and manifested in rehabilitation studies.

The role of PFs in the ICF classification is also linked to the ongoing discussion on the need for a full reconsideration of the ICF classification framework. An alternative ICF model in which medical health status is incorporated in PFs has been proposed (13). Moreover, Mitra and Shakespeare (14) proposed a visual scheme of the model in which environmental and personal factors are located at the top of the model, thereby emphasizing their importance. They also highlighted the importance of well-being, quality of life, and individual experience of agency when re-designing the ICF model. The need to review the ICF model is also shared by Sykes et al. (15), who suggested that any such process should be based on research evidence and, importantly, include people with disabilities. This ongoing discussion on if, and if so how, PFs should be included in the ICF indicates a need to systematically identify, analyze, and summarize how PFs have, to date, been studied in the field of rehabilitation.

In 2011, Geyh et al. (16) presented an overview of conceptualizations of the PFs component of the ICF. The review comprises 79 articles in which more than 200 concepts in total were labeled as PFs. Examples of the most significant of these include self-efficacy, attitudes, expectations, motivation, personality traits, and life goals. PFs were described in the articles as affecting disability and health and as having a significant role in the assessment of functioning and rehabilitation and in research and social security settings. The authors concluded that the PFs need to be standardized (16). In 2019, Geyh et al. (8) presented a classification of PFs. In this scoping review, we systematically collected research articles published after Geyh et al.'s work in (2011) (16) and applied the classification by Geyh et al. (8) in our analysis.

In 2017, the Finnish Rehabilitation Reform Committee submitted proposals for reforming Finland's rehabilitation services. Based on those proposals, the rehabilitation services reform was planned to take place between 2020 and 2022 as part of both a wider national reform program and as separate legislative projects. One important development area concerns the use of the ICF framework in organizing and producing rehabilitation services that meet the individual needs of the clients (4). The present review contributes to this reform work and aims, in particular, to provide a basis for determining the role of PFs in harmonizing monitoring systems and indicators of functioning. This review assembles research data and views on the need for the assessment of PFs and the possible need for a more precise classification as part of a comprehensive assessment of functioning. Our purpose was twofold: first, to summarize the PFs that have been investigated in research articles, irrespective of the study design, and second, to describe the reflections of the authors on the issue of PFs.

## Materials and Methods

### Study Design and Data Search

This study followed the scoping review methodological framework (17, 18). This method was appropriate, given the present objective of mapping the evidence on PFs. Literature searches were conducted by an expert information specialist in consultation with the research team. The search was undertaken in the following electronic databases: Cinahl, PubMed, ScienceDirect, and Sport Discus, and all potentially relevant studies published from 2010 to 2020 were extracted. The search terms were as follows: (ICF[Title/Abstract] OR “International Classification of Functioning”[Title/Abstract]) AND (personal[Title/Abstract] OR context^*^[Title/Abstract]). All study designs were eligible, whether qualitative, quantitative, or mixed methods. Methodology or guideline reports were also searched.

### Study Selection and Relevancy Rating

Throughout the selection process, the eligibility of studies was determined by applying established criteria: an article was included for the assessment of relevancy if it addressed one or more PFs in the context of ICF and excluded if it made no mention of PFs. Data selection was performed independently by two researchers. In addition, all members of the research team participated in the consensus discussions, in which the data selection protocol and choices were refined based on the inclusion and exclusion criteria. In the first step, the titles and abstracts were screened by two researchers.

The relevance of the full-text articles in relation to the research questions was then determined using the classification by Goodman et al. (19) (Table 1). Two researchers screened whether the article addressed one or more of the factors defined in the article as an ICF PF. Thereafter, articles were rated for relevance on a scale of one to six (1 = low relevance; 6 = high relevance). After the relevance ratings, only articles rated 5 and 6 were included in the further analysis.

**Table 1:** Relevance scale of the publication, adapted from Goodman et al. (19).

**Relevance**	**Definition**
**Included studies**	
6 = Directly and highly relevant (these studies are also included in class 5)	The abstract explicitly addresses PF. In the results section of the articles, PF are described in relation to the ICF classification (for example, as an outcome measure or factors affecting functioning). In addition, the role of PF in rehabilitation is reflected on in the discussion section.
5 = Definitely relevant	PF are mentioned in the abstract. In the results section of the articles PF are described in relation to the ICF classification (for example as an outcome measure or as a factor affecting functioning).
**Excluded studies**	
4 = Probably relevant	PF are mentioned in the abstract. The article does not distinguish which PF are defined as falling within the ICF classification.
3 = Possibly not relevant	The article mentions PF, but the focus on PF is not well articulated or consistently a focus throughout the paper.
2 = Probably not relevant	PF are mentioned in the abstract. Only a minor focus on PF.
1 = Definitely not relevant	The article makes no mention of PF.

### Data Analysis and Synthesis

Data extraction and analysis were conducted in two separate phases. The first phase of the data analysis included studies that reached level 5. Data on PFs were extracted, categorized according to the classification by Geyh et al. (8), and entered into a chart. The relevant descriptive characteristics of the studies (e.g., frequencies of methods used and study populations) were gathered and analyzed (see Tables 2, 3).

**Table 2:** Research designs of the included studies.

**Design**	**Number of studies** **Data *n* = 226** **Relevance level 5**	**Number of studies** **Data *n* = 51** **Relevance level 6**
Systematic or scoping review	39	9
Association of factors (for example regression analysis, latent class analysis)	72	14
Qualitative study (for example content analysis, phenomenological study, qualitative descriptive or case study)	48	10
ICF core set development and/or validation	20	11
Theoretical papers or recommendations	20	5
Quantitative descriptive/ cross-sectional study	14	3
Development and/or validation of measures	7	
Delphi study	4	
Other (Development of treatment, study protocol)	2	

**Table 3:** Target groups of the included studies.

**Target group**	**Number of studies** **Data *n* = 226** **(Relevance level 5)**	**Number of studies** **Data *n* = 51** **(Relevance level 6)**
Certain infectious and parasitic diseases (HIV)	1	
Endocrine, nutritional and metabolic diseases (diabetes mellitus, obesity, cystic fibrosis)	4	1
Neoplasms (cancer, pelvic chondrosarcoma)	10	3
Mental and behavioral disorders (mental disorder or illness, autism spectrum, attention deficit hyperactivity disorder, disorders of psychological development, cognitive impairment, transsexualism)	17	4
Diseases of the nervous system (multiple sclerosis, epilepsy, cerebral palsy, motor neurone disease, Parkinson's disease, complex regional pain syndrome)	23	
Diseases of the eye and adnexa (age-related vision loss)	1	
Diseases of the ear and mastoid process (benign paroxysmal positional vertigo, Meniere's disease, hearing loss or disability, tinnitus)	9	1
Diseases of the circulatory system (stroke)	21	5
Diseases of the respiratory system (chronic obstructive pulmonary disease)	1	1
Diseases of the musculoskeletal system and connective tissue (for example arthritis, rheumatoid arthritis, osteoporosis, neuropathic pain)	33	6
Diseases of the genitourinary system (pelvic organ prolapse)	1	
Congenital malformations, deformations and chromosomal abnormalities (Marfan syndrome, spina bifida)	2	
Symptoms, signs and abnormal clinical and laboratory findings, not elsewhere classified (apraxia of speech, aphasia, falls)	4	
Injury, poisoning and certain other consequences of external causes (for example brain injury, spinal cord injury, burn injury)	32	10
External causes of morbidity and mortality (lower limb amputation)	3	1
Factors influencing health status and contact with health services (occupational health, homeless people, wheelchair users)	8	
Disabilities, diseases and health conditions, unspecified (physical disabilities or impairments, chronic diseases or conditions)	33	11
Other (for example multilingual speakers, childhood development, special educational needs, older adults)	18	5
Theoretical (for example ICF children and youth version, PF classification development)	5	3

In the second phase, all the studies at level 5 that reached level 6 were extracted (see Table 1), and subjected to qualitative thematic analysis. All these studies included reflections on the role of PFs in rehabilitation. These reflections were subjected to a qualitative thematic analysis. The thematic analysis was implemented using a mind-mapping process in which the researchers analyzed qualitative themes identified in the reflections. Team members met frequently to compare mind maps and further consider their interpretations of the thematic categories and produce a thematic map of the findings. Thematic analysis was used to broaden knowledge on the role of PFs in rehabilitation research.

## Results

### Characteristics of the Articles

**Figure 1:**
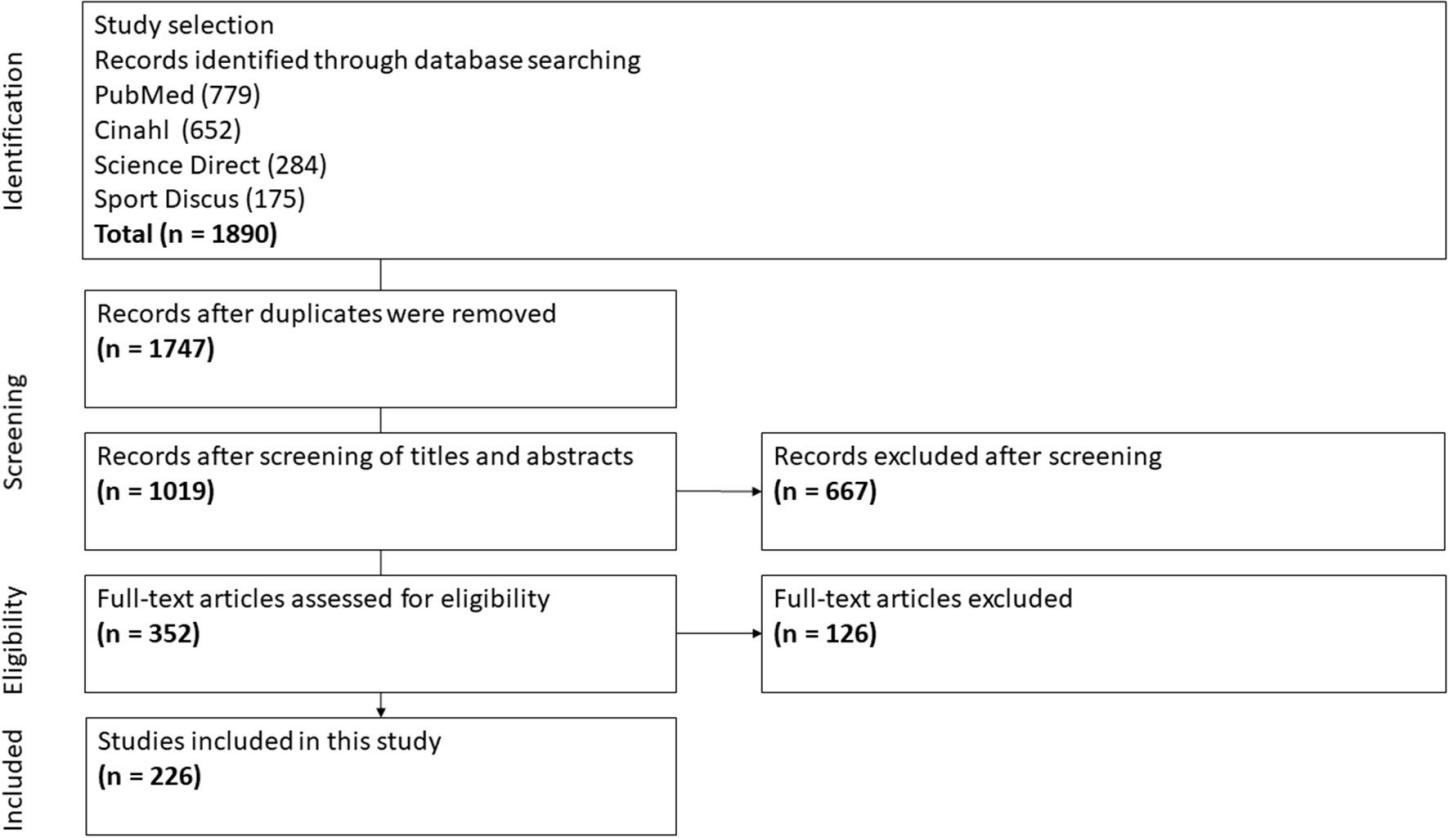
Flow chart of the study.

A total of 226 definitely relevant (level 5) research articles were included in the analysis. Of these, 51 articles were classified as direct and highly relevant (level 6), as the authors had reflected in the discussion section on the role of PFs in rehabilitation (Figure 1).

The research designs of the included articles ranged from quantitative, qualitative, and mixed methods to conceptual/theoretical (Table 2). The target groups of the articles were also heterogeneous, comprising different client groups and professionals (Table 3).

### Personal Factors in the Research Articles

The 226 articles addressed a wide variety of PFs. The PFs mentioned in articles, along with references to the articles in question, and factors included in the ICF as part of a component other than PFs or that do not describe functioning, are presented in Supplementary Table 1. The factors were linked to all seven of the categories defined by Geyh et al. (8). Of these articles, 154 articles (68%) addressed PFs that were linked to General patterns of experience and behavior (category 7). PFs related to Socio-demographic factors, most commonly gender and education (category 1) were addressed in 145 articles (64%), and factors related to Thoughts and beliefs (category 5), such as self-expectations and interest in various issues, in 106 articles (47%). PFs were also linked to the other four categories. PFs linked to Motives (category 6) were addressed the least, in only 25 articles (11%). Moreover, almost half of the studies (46%) dealt with PFs other than those listed in the classification of Geyh et al. (8). These included other diseases, quality of life, severity of injury, and compliance with treatment (Supplementary Table 1).

Factors included in the ICF as part of a component other than PFs or that do not describe functioning were mentioned as PFs in 71 articles (31%). For example, personality or personality traits related to ICF body functions (b126 temperament and personality functions) were defined as a PF in 17 articles, pain (b280–289) in 10 articles, and body mass index (b150 weight management functions) in 10 articles. Similarly, support from family, friends, or others was defined as a PF in 9 articles, although they are listed under environmental factors in the ICF (e3 support and interpersonal relationships). Factors that do not describe functioning but which were defined as PFs included lack of time, the ability of the therapist to communicate, and preparation for therapy.

### Roles of Personal Factors in Rehabilitation

**Figure 2:**
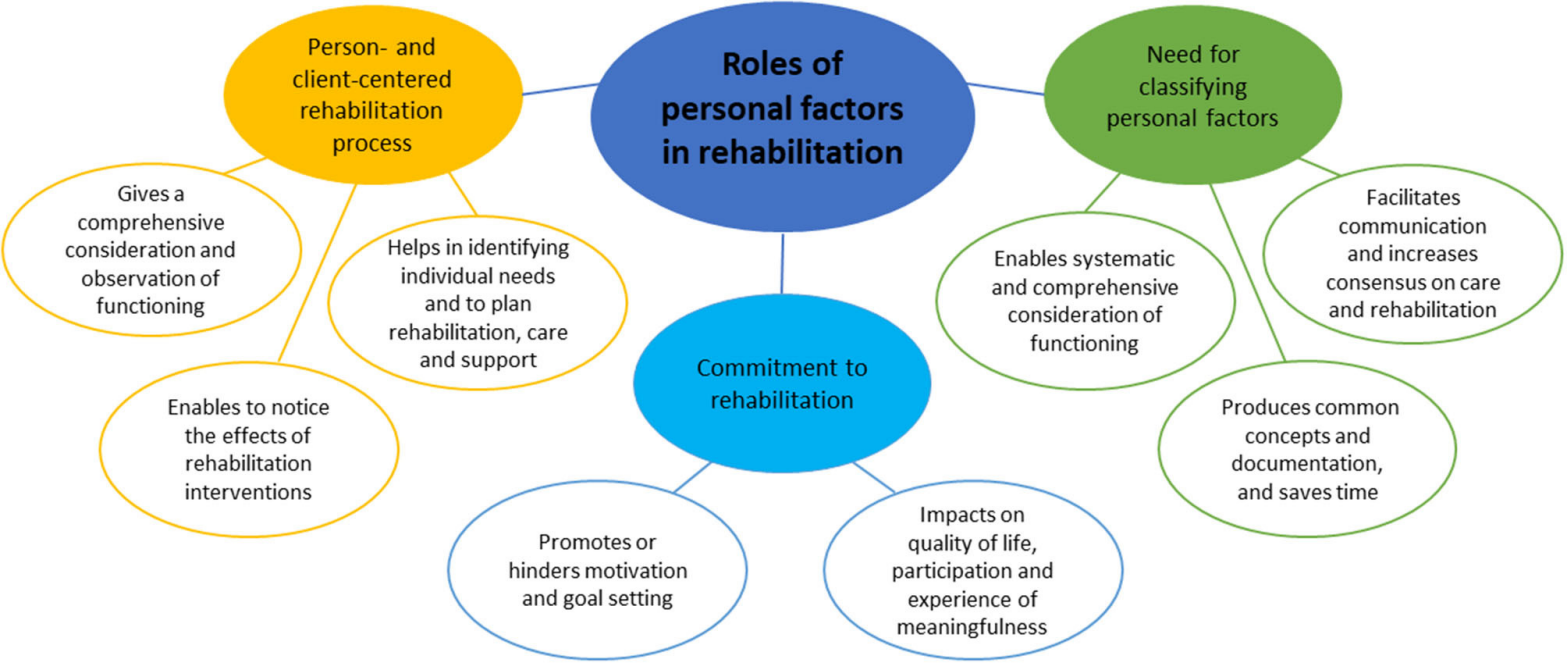
Roles of personal factors in rehabilitation.

The thematic analysis (of 51 articles) highlighted three themes on the role of PFs in rehabilitation: a person- and client-centered rehabilitation process, commitment to rehabilitation, and the need for classifying PFs (Figure 2). Each theme comprised different sub-themes.

#### Person- and Client-Centered Rehabilitation Process

In sum, the PFs reflected on in this group of articles largely concerned person- and client-centered care (20–39). PFs support a bio-psycho-social point of view of rehabilitation (40) and allow a comprehensive observation of functioning (8). In general, PFs were argued to be meaningful in person- and client-centered care (29). Professionals should permit the individual to drive the process (35). The role of PFs was reflected on in the different phases of rehabilitation. For example, the age and gender of a person seem to be especially important factors in rehabilitation planning (21, 23, 41–43). In addition, the classification of PFs helps in identifying the individually perceived needs of the clients and the planning of individual care (i.e., medication) (44). It also helps professionals to plan and select rehabilitation interventions for clients (45) and enables them to see the effects of these interventions (31). PFs seem to be more relevant to physical activity and training than environmental factors (38). A PF may act as a significant enabler or deterrent in determining the social, medical, or rehabilitative benefits sought (46). Consideration of PFs can have an impact on multiple outcomes, including quality of life (24, 32, 47), functioning and participation in society (32), and social integration (48).

#### Commitment to Rehabilitation

Several articles discussed PFs around the theme “commitment to rehabilitation.” In particular, the role of PFs in motivating rehabilitation was addressed in many articles. Hawkins et al. (25) argued that PFs should be taken into account when planning rehabilitation as they are relevant to motivation. It was also argued that PFs can promote or hinder the motivation of a person (30), device uptake (49), return to work (50) and realistic goal setting in rehabilitation (27). Giving consideration to personal interests generates resources and prevents frustration with rehabilitation, and thus promotes the commitment of a client (51). Positive coping strategies, self-efficacy, and an optimistic attitude toward rehabilitation are important factors in its success (33, 52, 53). They also allow us to understand and take note of the experience of illness and satisfaction by the clients with their activities (54). Willingness and an optimistic attitude toward rehabilitation are thus meaningful factors (30). The confidence of a person in his/her own abilities and capacities is also important in promoting commitment to the rehabilitation process (51). PFs can also help in identifying the challenges that rehabilitation presents to individuals (31).

#### Need for Classifying Personal Factors

The need for a classification of PFs was considered in the articles from different perspectives. Generally, it was stated that professionals should recognize the benefits of having a classification of PFs (47, 55), notably in the context of rehabilitation (56). For example, a classification would help interprofessional teams to achieve a consensus on care and rehabilitation (33). Furthermore, a classification would facilitate a comprehensive and systematic examination, description, and documentation of problems and resources of a client and how these impact on functioning (55, 57) and would also save time (46). Making assessments and decisions based on the use of non-standardized individual factors involves high risks (46). The systematic gathering of data can assist in planning and implementing more precisely targeted interventions and in monitoring rehabilitation outcomes (31). A standardized classification could also help professionals to develop common concepts and documentation (12, 58). It was also argued that non-standardized use presents a risk in rehabilitation (8). Without the inclusion of PFs, the model of functioning remains narrow and reduces the status of an individual to one of illness and disability bereft of autonomy, subjectivity, and humanity, and thus ignores the whole life context of the individual. Without PFs, the ICF is an unhumanized model (8, 12, 40).

## Discussion

This scoping review summarized the literature on research that included discussion of ICF PFs to better understand what PFs are and to analyze their role in rehabilitation. As in the previous review by Geyh et al. (16), the studies included in this review were heterogeneous in their research settings, target groups, and targeted stage of the rehabilitation process. Mentions of PFs were extracted from all the eligible studies and, excepting those that were clearly not PFs, grouped into seven categories according to Geyh et al. (8). PFs were most often linked to personal experiences or habits, sociodemographic factors, and personal thoughts and beliefs. The qualitative analysis of the importance and meaningfulness of PF in rehabilitation yielded three themes: a person- and client-centered rehabilitation process, commitment to rehabilitation, and the need for classifying PFs. Armed with these findings from recent research studies, we entered the debate on the role of PFs in rehabilitation, their importance in understanding functioning and disability, and their ethical use (11, 15).

### Personal Factors in Rehabilitation Studies

Overall, the studies revealed a wide range of different types of PFs. While the included studies used heterogeneous methods and focused on different target groups, they all considered PFs to be important factors in assessing functioning and in planning and implementing rehabilitation. While all the included studies (*n* = 226) included an analysis of PFs, they were not always the central aim. In fact, only a quarter of the included studies (*n* = 51) focused on PFs to the extent of explicitly drawing conclusions about them, and only 14 studies called for the classification of PFs.

Martinuzzi et al. (59) argued for the importance of adding PFs described by clients to those that are already described in classifications. The same PFs were mentioned in different types of research studies, thereby indicating how essential they are for understanding situations from the perspective of a client. Surprisingly, however, the PFs named in many studies were clearly not PFs and could be linked to some of the existing ICF components. A possible explanation for this is that the ICF is still not thoroughly understood with respect to which factors belong to which components. Alternatively, the short descriptions given about the PFs in the ICF may not be clear enough for users. These results are in line with those of Martinuzzi et al. (59), who emphasized the need to understand the whole ICF model, including the relations between its components, when assessing PFs. However, it can be also argued that the ICF itself is ambiguous. In particular, factors such as personality or motivation, that can be linked to the ICF b1 mental functions category and linked to the ICF as PFs in the studies included in this review, showed that these constructs merit consideration when further developing the ICF.

### Roles of Personal Factors in Rehabilitation

Our thematic analysis showed that PFs play an essential role in rehabilitation. Three different themes on their role emerged. The first theme concerned their role in supporting a person- and client-centered rehabilitation process. Assessment of PFs is essential when planning rehabilitation and when documenting information on functioning. Asking and understanding about PFs can foster core components of person- and client centered rehabilitation such as respect for values, beliefs, experience, and contexts, and inclusion of family as defined by the client (60). It has also been argued that person-centered care could have a positive effect on rehabilitation outcomes, although it has not yet been fully implemented in rehabilitation settings (61). The rehabilitation process combines two theoretical frameworks: treatment theory, which provides tools on how a change in a particular factor can be brought about, and enablement theory, which acknowledges that functioning is complex and determined by multiple factors, and which seeks to model these complex interrelationships (62). To apply enablement theory in the rehabilitation process, it is essential to understand individual variation in PFs. Our results show that PFs contribute essential information that should be linked with information on functioning in the rehabilitation process of a person. However, in clinical practice professionals mostly document them in the history of a client in a narrative form. Using unified terminology could enhance documentation quality, but this does not necessarily mean that all PFs should be contained in a single classification.

The second theme highlighted the importance of PFs for the commitment of a person in various rehabilitation programs and in different phases of rehabilitation. Motivation is clearly a personal matter, and it has been noticed to be an important predictor of adherence to, for example, exercise interventions (63) In addition, it is important to take into account that different clients consider different things important, as this affects commitment. Similarly, the need, highlighted by Lee et al. (64), to recognize the experience of purposefulness by a client influences rehabilitation outcomes. Professionals can learn how to support empowerment and strengths of a person by considering how various PFs might facilitate or hinder the commitment of a person. These findings support previous studies that have suggested reorganizing the ICF model to emphasize PFs (13, 14). Notably, we found no mention of the concern that a classification of PF within the ICF could lead to “blaming” the person for their functional limitations (11) in any of the studies. Instead, PFs were invariably used to support clients in their rehabilitation process.

The third theme concerned the importance of classifying PFs for the benefit of professionals. Studies supporting this idea identified the need to develop the ICF classification and its core lists to include PFs. This would create a comprehensive and systematic tool to facilitate communication, increase consensus, and save time. Another question concerned whether a minimum generic list of essential PFs could be developed for use in clinical practice with all clients. Clinically, the ICF can be used to organize and code the assessment data on functioning and environmental factors. As the PFs of the client can have a strong influence not only on health and functioning but also on the rehabilitation process, professionals would benefit from reliable tools to help in the assessment and guide the discussion. Such a tool could be, for example, a minimum list of potentially important PFs. In client-centered practice, the professional should, together with the client, consider which factors are important and relevant for that client and use this knowledge to discuss how best to help the client go forward in the rehabilitation process (65). Future research should evaluate whether this would enhance core elements of client-centered rehabilitation, such as communication and partnership (66). It seems that in the absence of a generally accepted classification, several differing classifications have arisen (10, 67). Based on this scoping review, the classification proposed by Geyh et al. (8) covers a lot of important PFs of relevance for client-centered rehabilitation. However, a large number of PFs were not included in the Geyh et al.'s (8) classification. This must be borne in mind when applying the classification in clinical practice. Since the completion of the present analysis, Grotkamp et al. (68) published a classification that includes PFs more broadly related to, for example, life situation and physical functioning compared with Geyh's classification. It would therefore be useful to apply them as complementary classifications when assessing functioning in relation to PFs in clinical practice.

### Research: Clinical and Ethical Implications

This review did not seek an answer to the question of whether to classify PFs or not. All the included studies stated that they are important, while a few proposed classifying them. However, a complete taxonomy or classification of all possible PFs may not be necessary as some of them are already included in other classifications or instruments. Many information structures in health and social care include PFs, particularly factors in categories 1 and 2 of the classification by Geyh et al. (8), such as gender, age, occupation, or education. In Finland, the National Code Server has defined some common information components to unify documentation of the same type of data using the same structures. These components include PFs related to life habits (category 7), such as motion, nutrition, sleep/rest, as well as smoking and alcohol use habits (69). Rehabilitation professionals also use instruments that focus on PFs and structurally assess PFs based on the subjective experience of clients. For example, the Occupational Performance History Interview (OPHI-II), a method that collects unique data on a person's functional history during working age (70) can be subsumed under personal history (category 3). PFs regarding health, feelings, and mood of the self (category 4) and attitudes, expectations, and motives (categories 5 and 6) of the self can either be discussed freely with the client or incorporated in a structured interview, using, for example, the Readiness for Return to Work Questionnaire (71) or the relevant part of the Model of Human Occupation Screening Tool (MOHOST), which assesses the own will and motivation of the client (72). Future research should explore precisely what instruments or other methods of PF are available and whether they are comprehensive enough to describe and document the wide variety of PFs.

All the public health care institutions of the Nordic countries subscribe to a democratic value system, in which all citizens have equal rights to individualized and person-centered health care services (73). In many countries, the professional use of PFs is guided by legislation and other principles. For example, the UN Convention on the Rights of Persons with Disabilities (74) stipulates that all disabled people should be treated equally. The new EU legislation takes this one step further and considers a client's personal data, such as functioning or PFs, as sensitive data (75). In the EU, at least, this gives clients better protection and control over their personal information and how this information is used in rehabilitation processes. Moreover, health care professionals are under a duty to base their decisions and actions on ethical principles. These include empathy, honesty, and confidentiality. Finnish physical therapists, for example, should adhere to the basic ethical principles of doing good, avoiding bad actions, and respecting client autonomy and justice (76).

### Strength and Limitations

A key strength of this study was the implementation of a rigorous and systematic methodological approach. Furthermore, by addressing the importance of PFs in rehabilitation research and practice, this study may be of value in the future development and use of the ICF classification.

This scoping review synthesized the key characteristics attributed to PFs in the rehabilitation literature. Due to the broad focus of the study, we may have failed to identify all the relevant studies. However, consultation with an information specialist throughout the search process reduced the likelihood of this limitation. To enhance the trustworthiness of the data, the team members cross-checked and verified the search results in pairs. Owing to the scoping review method (77), the methodological quality or risk for bias of the included articles was not evaluated. Moreover, this study does not produce a critically appraised answer to the question of whether PFs should be classified. The broad aim of the review generated a large number of references. More specific inclusion and exclusion criteria might have enabled a more precise focus on the role of PFs in rehabilitation.

### Conclusions

A substantial number of studies concluded that PFs have an important role and a specific meaning in rehabilitation processes. PFs foreground the principle of person- and client-centeredness in such processes. Furthermore, when PFs are well understood and taken into account in assessing the functioning of a client, the professional will have a better understanding of how to strengthen the commitment of a client. Professionals would also benefit from a classification of PFs to facilitate systematic documentation and save time. Future research should define what tools to use and what factors to include in a list of the minimum PFs needed to guide rehabilitation processes. In the meantime, it is recommended to use the ICF framework as an instrument for the structuring of information and concepts related to functioning, even if PFs have not been further defined at the level of categories. The classification developed by Geyh et al. (8) and/or that by Grotkamp et al. (68) can serve as checklists when mapping, together with the client, which PFs promote or hinder activity and participation, and how important different factors are to the client.

## Author Contributions

MK, AK, SS, HA, EX, and TH: Conceptualization. MK, HA, and AK: Theoretical framework and literature review. MK: Project administration. MK, AK, SS, EX, TH, and HA: Analysis and writing and editing. All authors have read and agreed to the published version of the manuscript.

## Conflict of Interest

The authors declare that the research was conducted in the absence of any commercial or financial relationships that could be construed as a potential conflict of interest. The handling Editor declared a past co-authorship with one of the authors HA.

## Publisher's Note

All claims expressed in this article are solely those of the authors and do not necessarily represent those of their affiliated organizations, or those of the publisher, the editors and the reviewers. Any product that may be evaluated in this article, or claim that may be made by its manufacturer, is not guaranteed or endorsed by the publisher.
