# Supplementary material for: ICF Personal Factors Strengthen Commitment to Person-Centered Rehabilitation – A Scoping Review
Source: Front Rehabil Sci. 2021 Aug 16;2:709682. doi: 10.3389/fresc.2021.709682 (PMC9397796; doi:10.3389/fresc.2021.709682)
Supplement: Supplementary file 1 [file Table_1.DOCX]

**Supplementary table 1. Personal factors in included articles**

| **First author Year** | **Personal factors in included articles**  (Factors included in the ICF as part of a component other than personal factors or that do not describe functioning) | **PF Categories defined by Geyh et al. 2019 = catogories 1 - 7 Other PF = category 8** |
| --- | --- | --- |
| Abeysekara 2014* | Gender, age, coping skills, social and family background, educational level, life experiences, emotional and learning styles, eating habits, (cognition) | 1, 2, 3, 7 |
| Alcantara  2018 | Sex, age groups, number of children, marital status, familiar income, education, smokes, consumes alcohol, physical activity, comorbidies | 1, 2, 7, 8 |
| Amano  2019* | Age, gender, education level, marital status, race/ethnicity, income, (personality: neuroticism, extraversion, openness, agreeableness, conscientiousness) | 1, 2 |
| Amatya  2017 | Attitude, coping ability, educational background, socio-economical background | 1, 5, 7 |
| Amster  2016* | Age, educational level, ethnicity, gender, fear of rejection, self-image,valuing relationships and being valued, accepting help,self-presentation, high social self-monitoring, faith, helpful cognitive schemas, lack of social skill, ot discussing disability, good interpersonal skills, adaptation over time, effort, proactive, communication, patience and acceptance, anxiety, attachment style, broadening personal definition of sexuality, gaining sexual experience, decreased sexual interest, culture, bitterness after breakups, social discomfort, not proactive, opting out, (cognitive ability) | 1, 7, 8 |
| Andelic  2012* | Age, gender and pre-injury employment status | 1 |
| Andresen 2015* | Perspective of what it means for communication to be ‘‘easy’’, attitude, motivation, (the individual’s personality traits) | 5, 7, 8 |
| Arendde  2015 | Individual background, e.g. gender, race | 1 |
| Arnodottir 2011* | Leisure time activity, household physical activity, work-related physical activity, medical diagnosis, age, gender | 1, 7,8 |
| Arnold  2012* | Age, lifestyle habits, gender, coping styles, well-being, self-regulatory efficacy, (pain) | 1, 5, 7, 8 |
| Awad  2013* | Self-confidence on one’s own efficiency and fitness, education-lifestyle disease specific management, self-efficacy, physical inactivity, sedentary lifestyle | 5, 7 |
| Ayvat  2019 | Age, gender, duration of the disease, the number of falls during the past 6 months, (BMI) | 1, 3, 8 |
| Baert  2012* | Age, gender, stroke characteristics (type of stroke, stroke area and side of lesion), the National Institutes of Health Stroke Scale and Barthel Index as indicators of initial stroke severity | 1, 8 |
| Bagraith  2013 | Background demographics, self-efficacy, coping styles/strategies, understanding of or beliefs about, index health condition, physical activity/level of fitness, socioeconomic status, educational status, co-morbidities, professional/work history, habits, behaviour pattern or character style, upbringing, past and current life experiences, current (non-medicinal) substance use | 1, 3, 5, 7, 8 |
| Baird  2010 | goal attainment | 6 |
| Barclay  2014 | Secondary health conditions, locus of control or frustration with choice and control over important matters in their life,  age, gender, ethnicity, anxiety, injury type, (pain) | 1, 4, 5, 8 |
| Batten  2020* | Challenges adjusting to change/being able to adjust to change, having a positive attitude, goal setting and a purpose for community walking | 5, 6, 7 |
| Bayly  2016 | Wanting to live as usual, not be a burden maintain independence, though experienced uncertainty, hope, thoughts of death, sadness, shame and guilt, asking for help, a respiratory symptom distress cluster comprising of cough, breathlessness and fatigue, and compounded by distress and associated meanings (corresponding to WHO-ICF personal factors), impacted on activities and participation in daily life, beliefs, behaviours, the symptom burden, strive to maintain normality and independence in everyday life activities and fear being a burden to their families, personal beliefs an optimistic perspective, positive self-presentation and normalisation as coping strategies | 5, 6, 7, 8 |
| Beaudry  2019 | Confidence in their abilities, feeling of well-being | 4, 5 |
| Becker  2010* | Age, level of education, family disruption, self esteem, self-blame, coping, resilience, tabacco abuse, alcohol abuse, drug abuse, life-style, loss of independence, Personality change, existential concerns, depression, anxiety disorders, adjustment disorders, loss of role, social withdrawal, dignity, loneliness, psychiatric disease, personality disorders, medical history, suicidality, stigmatization, long hospital stays, medical care during psychological session, (shame, hopelessness, guilt, helplessness, emotional and practical support for the family, only few psychologist have expereience in the treatment of patients with head and neck cancer) | 1, 2, 5, 7, 8 |
| Berzina  2013 | age, gender, place of residence, time since onset of stroke | 1, 3 |
| Bilgin  2019 | Age, gender, educational level, fear of movement, symptoms of depression and anxiety, working duration | 1, 3, 4 |
| Blake 2019* | Sex, race, age, education, attitude, profession, habits | 1, 5, 7 |
| Bogart 2018 | Age, ethnicity, gender, income, self-esteem | 1, 7 |
| Bogart 2017* | Age, ethnicity, gender, income, psychological distress | 1, 4 |
| Boldt 2012 | Gender, age, patient education, profession, acceptance of life in wheelchair, acceptance of new sexuality, acceptance of new identity, information for the patient, knowledge deficit about SCI, patient attitude, perceived low options, adjustment to new body image, adaptation to new life style, coping with everyday life, patient strategy to survive, coping with grief, denial, difficult behavior, perceived adjustment in familial roles, perceived adjustment in societal roles, loss of status, patient does not want to return home, patient feels secure in the hospital, patient teaching | 1, 5, 6, 7, 8 |
| Boogaard  2013 | Age, gender, life satisfaction, role functioning, personality disorder, catastrophizing at baseline, anxiety, depression, disease conviction, affective distress | 1, 7, 8 |
| Boonen 2010* | Education, positive beliefs, adjust to the disease, optimism, accepting, adapting (such as finding creative solutions), gaining insight into the disease, trust in health professionals, adaptive coping strategies in the experience of health | 1, 5, 7 |
| Bornbaum 2013* | Age, gender, race, socioeconomic status, habits, lifestyle, choices, health behaviors, health condition, comorbidities | 1, 7, 8 |
| Bossmann 2011* | Fear-avoidance beliefs, self-efficacy, coping styles, health conditions such as obesity and osteoporosis, (psychosocial factors ) | 5, 7, 8 |
| Bours  2016 | Age, gender, ethnicity, cancer-threat appraisal, faith and meaning/peace, personal lifestyle behavior, denial, optimism, sense of coherence, (the personality of CRC survivors) | 1, 5, 7, 8 |
| Brandt  2019 | Age, menopausal status, depressive symptoms and poor emoonal state, co-morbidies such as cardiovascular disease and hypothyroidism, history of mulple normal deliveries and previous surgery | 1, 4, 8 |
| Britto  2018 | Age, gender, race, economic conditions, level of education, social background, employability, different ways of facing problems, life course adversities, lifestyle, (attendance to medical appointments or hospitalisation) | 1, 3, 7 |
| Bruls  2018 | Gender, age, educational attainment, work status, marital status, participants’ opinion regarding cause, fear avoidance beliefs, (BMI Body Mass Index) | 1, 2, 5 |
| Brutt  2016* | Age, gender, employment, social background, awareness of personal limitations, acceptance of personal limitations, self-reflection, including health promotion and stress management, dealing with mental illness and (self)-stigmatization, lifestyle (personal), interpersonal skills such as openness, (preparation before psychotherapy) | 1, 5, 7 |
| Burns  2015 | Emotional impact, loss of autonomy (independence and privacy), physical experience | 5, 7, 8 |
| Chan  2016 | Gender, race, age, education, disability type, significant disability, benefits cash/medical | 1, 8 |
| Chang  2013 | Age, gender, education, race, income, relationship, duration of homelessness | 1, 2, 3 |
| Chase  2018 | Age, gender, race/ethnicity, ducation, marital status | 1, 2 |
| Chiu  2017* | Age, gender, working status, disability certification, severity of disability | 1, 8 |
| Chiu 2019 | Gender, race, ethnicity, age, drug and alcohol use, use of ARV medications, mental health diagnosis, mental health treatment status | 1, 7, 8 |
| Choukou_2019 | Work, occupation perception, preference, (costs) | 1, 5 |
| Cimarolli 2017 | Age, gender, race | 1 |
| Congdon  2010 | Age, co-morbidities, pre-morbid health status, (psychological status/personality (motivated), intact cognition) | 1, 8 |
| Cornelius  2011 | Age, gender, education, return-to-work, socioeconomic status, sole breadwinner, history of previous sickness absence, recovery expectation | 1, 2, 3, 5 |
| Covington 2020 | Expectations of treatment, medications, (cancer related distress) | 5, 8 |
| Culler  2011 | Poor insight to deficts, poor safety awareness, poor adjustment to disability, refusal to use strategies and or accommodations, length of time since worked, premorbid employment history and position in company, not seeing self as able to work, poor work performance pre onset, dislike for prior job and or employer, low motivation for return to work, lack of high school diploma/GED, need for surgeries, depression | 1, 3, 6, 7, 8 |
| DeBoer 2010 | Age, gender, (intellectual impariment, intellectual impariment: body structures and functions, activity limitations, and participation restriction) | 1 |
| De Beer 2014 | **General personal data/socio-demographic factors:** age, gender, language, socio-economic status, education; accepting the LD/dyslexia, experiences with LD/dyslexia, impact of the LD/dyslexia, job/career satisfaction;  **General ‘mental’ personal factors:** aggravation, common sense, confidence, creative, demoralized, desire to help others, detail oriented, diligent, empathic, humorous, innovative, learning& coping strategies, literacy level, openness, patience, personal problems, pride, satisfaction with life, secretive, sensitive to emotional experiences of others, self-advocacy, self-control, self-disclosure, self-efficacy, self-empowerment, self-esteem, self-promotion, self-reliant, self-sufficient, social person, stress, locus of control, management of the LD/dyslexia; avoidance, stress-experience/being stressed/stubborn/tolerant/visual | 1, 5, 7, 8 |
| de Rooij 2019 | Feelings of being watched, overestimation of own limits, dislike walking, loving nature, fresh air or tranquility, decreased initiative or lack of a purpose to go for a walk, anxiety and insecurity, motivation, good planning skills, active personality, fulfillment, comorbidities, stress | 4, 6, 7, 8 |
| de Kloet 2015 | gender, race/ethnicity, age at onset, time since onset, problems in pre-injury adaptive functioning, general health problems | 1, 3, 7, 8 |
| Delle Fave 2017 | Employment status, education level, level of disability | 1, 8 |
| Dempsey 2010 | Gender, family income, maternal education, marital breakdown, preference for active pursuits, coping style, lifestyle, emotional content of parent-child conversations, (teacher expectations, temperament, peer acceptance) | 1, 2, 5, 7 |
| den Ouden 2013 | Age, gender, education, self-management abilities, anxiety/panic disorders, depressive symptoms, smoking,  quality of life | 1, 4, 7, 8 |
| Deramore-Denver 2017 | Mood, interest, use of compensatory strategies | 4, 5, 7 |
| de Schinner 2016 | Gender differences, honesty, loyalty, creative talents, attention to detail, personal factors e.g. self-esteem, restricted interests | 1, 8 |
| Ditchman 2016* | Internal psychosocial states, and characteristics, age, coping style, past experiences, gender, sex, disability acceptance, social self-efficacy | 1, 3, 7 |
| Dorstyn 2017 | Mood and affect, attitudes and beliefs, attitude towards the biopsychosocial impact of MS, self-efficacy, coping ability, (fatigue) | 4, 5, 7 |
| Dur 2015 | Job satisfaction, sense of coherence and social appreciation, own attitudes, reflecting about one’s life in an optimistic way, meaningful activities for the individual and/or the societal context, adaptation to changed living conditions, coping, eating habits and weight concerns, resilience, self-efficacy, involvement into disease management | 5, 7, 8 |
| Dutra 2017 | Gender, age, schooling, occupation, present work situation, income, relationship status, number of children; life habits: physical activity levels, smoking habits, patterns of alcohol intake | 1, 2, 7 |
| Dwyer 2015* | Feeling isolated, loss of camaraderie on discharge home, loss of shared experience, loss of peer support, loss of sense of belonging, loss of safety/security, feeling of inferiority, not psychologically prepared for returning home/anxiety, depression, adjusting SCI/coping, invasion of privacy/feeling exposed, self-management, lack of control, loss of independence, adjustment to SCI, rebuild/reframe life, re-establish self, coming to terms with changed self, hope for recovery, positive attitude, sense of importance/being valued, sense of belonging, self-management sense of control/locus of control, validation/endorsement, confidence to translate skills to new environment “can do it however you want", peer modelling/mentoring, importance of independence | 4, 7, 8 |
| Earde 2018* | Frustration, interest, willingness and self-acceptance, self-efficacy, motivation, (being an introvert) | 4, 5, 7 |
| Ellis 2011 | Age, gender, education, employment status, household income, exercise knowledge, motivation, self-efficacy | 1, 5, 7 |
| Escorpizo 2010* | Age, gender, race and ethnicity, religion, educational level, beliefs, behavior, habits, height, weight, language level, culture, handedness, custom | 1, 5, 7, 8 |
| Esmail 2020 | Gender, self-esteem, public self-consciousness, self-expression, confidence , accomplishment, appearance normalization, (psychological comfort, culture) | 1, 5, 7, 8 |
| European Physical and Rehabilitation Medicine Bodies Alliance 2018 | Motivation, motives, self-assurance, assertiveness | 6, 7 |
| Fannin 2016 | Gender, race, age, educational level, lifestyle, being labeled “disabled” | 1, 7, 8 |
| Farber 2015 | Purpose in life, environmental mastery, self-acceptance | 6, 7 |
| Fekete 2012 | **Socio-demographic personal characteristics:** age, gender, occupation, education, income, ethnicity; **position in the immediate social and physical context:** marital status; **feelings:** enjoyment, dislike, embarrassment, feelings of incomple dissatisfaction with the body, fear of injury/ poorer condition, fear of health complications due to low PA; **thoughts and beliefs:** perceived disease risk, lack of knowledge, acquiring new knowledge, lack of motivation, goal setting, improving physical appearance, self-image, decreases feelings of being disabled, improving self-esteem, self-efficacy to participate in PA, intentions to engage in physical activity, attitudes, subjective norms and perceived behavioral control; **patterns of experienceand behavior:** level of PA in youth, pre-injury level of PA, lack of time, learning new physical strategies, routines, recalling new experiences to exposure, accept narrower body margins, learning to read the body, being a role model | 1, 2, 4, 5, 7 |
| Finger 2017 | Gender, ethnicity, education, socioeconomic status, financial situation, family living situation, family/work relationship, major life events, medical history, failure of previous drugs, mood, fear of side effects, health expectations, health/illness beliefs, readiness to change, assessment experience, lifestyle factors, physical activity, diet, smoking, alcohol, self-management, motivation, resielience/adaptability, adherence to treatment, compliance/coping, health literacy, nutrition/food security, comorbidities, depression, anxiety, regional effect on placebo response (obesity, BMI, social support, social isolation, psychological distress) | 1, 2, 3, 4, 5, 7, 8 |
| Finger 2016 | Age, gender, nationality, citizenship and ethnicity, language, educational background, occupational background, economical background, position in family, position in partnership and marriage, position in an informal social context, position in a formal social context, position in housing context, life events, biographical course emotions, moods, knowledge and concepts, personal memories, personal attitudes, personal beliefs, personal values and norms, personal evaluations, personal preferences, imagination, fantasy and dreams, personal needs, personal interests, personal goals, patterns of feelings and handling feelings, patterns of thoughts and handling thoughts, pattern of motives and handling motives, patterns of behaviours and handling behaviours | 1, 2, 3, 4, 5, 6, 7 |
| Francescutti 2011 | Socio-demographic information, person perception and evaluation of current needs, (diagnosis considered as a proxy for health conditions) | 1, 6 |
| Fulcher 2015 | Age, gender, race, languages spoken | 1 |
| Gailey 2020 | Age, time since amputation, associated comorbidities, level of amputation | 1, 3, 8 |
| Gan 2014 | Age levels, educational placement, mother’s educational level, family income | 1 |
| Garner 2013 | Psychiatric comorbidities, learning disabilities | 8 |
| Gass 2019 | Coping strategies | 7 |
| Geidl 2014* | Knowledge, risk perception, outcome expectations, outcome experiences, goal intention, self-efficacy, coping strategies, self-concordance, action control, (implementation planning) | 5, 6, 7 |
| Geyh 2011* | Coping styles, character style, behavior patterns,emotional experience, age, gender, ethnicity, race, martital status, acceptance, adjustment, avoidance, denial, lifestyle factors: nutrition, smoking, alcohol exercise | 1, 2, 4, 7 |
| Ginis 2012 | Age, gender, years post-injury, perceived health status, perceived behavioral control, subjective norms, attitudes,  intentions barrier self-efficacy | 1, 3, 5, 7 |
| Glaessel 2011 | Feelings of shame, knowledge of disease, lack of knowledge, apprehension regarding effects of further treatment, motivation, coping strategies,self-management strategies, lymphoedema management, scar management, pain management | 4, 5, 7, 8 |
| Glocker 2012* | Work situation, living situation, education, profession, family status, spirituality, satisfaction with job, perceiving oneself as victim, expectations from medical services and health systems, poor perceived exterior circumstances, self acceptance, acceptance of LBP, lifestyle/ignorance of healthy lifestyle/sedentary lifestyle, coping, avoidant behavior, general behavior, ignorance of LBP, psychological morbidity, concomitant diseases, body weight, compliance, general health, physical fitness, cognitive resources | 1, 2, 4, 5, 7, 8 |
| Glässel 2011 | Age, gender, education, autonomy, expectations to oneself, individual coping strategies, personal risk factors | 1, 5, 7, 8 |
| Glässel 2011 | Illness knowledge, optimistic/positive attitude, self-concept, self-perception, autonomy/independence, self-management, life values, life goals, lifestyle, coping, sense of mastery, endurance/discipline, hardiness, problems/ worries/uncertainty, compliance, (brain plasticity and recovery) | 5, 7, 8 |
| Gomes 2019 | Age, gender, (body mass index BMI) | 1 |
| Gradinger 2011 | Differentiate between free time and work, the aspect of visibility of sleep disorders, (ensuing problems in social interactions) | 7, 8 |
| Grotkamp 2020 | **Age:** calendar age, psychosocial age, biological age, age otherwise specified, age unspecified; **gender:** biological sex, social gender (gender), gender otherwise specified, gender unspecified, general characteristics of a person otherwise specified, general characteristics unspecified; **physical factors:** body measurements, body shape and body composition, movement related factors, cardiovascular and respiratory factors, factors of metabolism, factors of the sense organs, physical factors otherwise specified, physical factors unspecified; **personality factors:** extraversion, factors of emotionality, conscientiousness, openness to new experiences, sociability, optimism,; **cognitive and mnestic factors:** factors of intelligence, cognitive factors, mnestic factors, cognitive and mnestic factors other specified, cognitive and mnestic factors no details designated, mental factors otherwise specified, mental factors unspecified; **attitudes:** worldview, attitude towards oneself, life satisfaction, attitudes towards health, illness and disability, attitude towards support from other people, attitude to interventions and technical assistance, attitude to financial insurance and utility services, attitude towards education, attitude towards work, attitude to social life and society, attitudes otherwise specified, attitudes unspecified; **action competence:** social skills, language proficiency, methodological competence, self-competence (empowerment), expertise, media literacy, action competence otherwise designated, competence to act unspecified; **habits:** eating habits, habits when consuming luxury foods, exercise habits, habits in everyday routines, leisure habits, sexual habits, communication habits, hygiene habits, habits of dealing with money and material goods, behavioral habits other specified, behavioral habits unspecified, attitudes, action skills and habits otherwise specified, attitudes, competence and habits unspecified; **situation in life:** family status, status in immediate and wider social context, housing situation, employment status, economic status, legal status, social status, cultural status, belonging to ethnic groups, educational status, situation in life otherwise specified, situation in life unspecified (personality factors: personality factors other specified, factors of personality unspecified) | 1, 2, 5, 7, 8 |
| Grunt 2013 | Age, previous surgery, cooperation and motivation, (diagnosis, follow-up therapy) | 1, 3, 7 |
| Hamed 2012 | Social background, concerns and feelings, psychological assets, self-esteem and well-being, coping styles, habits, weaknesses | 1, 5, 7, 8 |
| Hancock 2017* | Age, race, education, occupation status, marital status, significant health concerns, history of gender transition: age at transition, time since transition, or stress from general life upheaval surrounding transition | 1, 2, 3, 8 |
| Hand 2016 | Maladaptive behavior, socialization coping skills, somatic complaints, (nonverbal IQ, attention problems, emotional reactivity) | 7, 8 |
| Hansen 2017 | Sense of coherence | 7 |
| Hardy 2013 | Profession, education, previous marriage, biological parenthood, age when aware of gender dysphoria,  fear of failure or lacks confidence, cultural or religious beliefs, upbringing, outlook | 1, 2, 3, 4, 5, 7, 8 |
| Hassett 2011* | Age, sex, pre-injury occupation (high skill, low), exercise history (style/frequency) | 1, 3 |
| Hawkins 2015* | Self-efficacy | 7 |
| Heerkens 2017 | **Sociodemographic factors/general personal data (facts) sociodemographic factors:** age, country of birth, education/educational status, ethnicity, gender, language, nationality, religious affiliation, socio-cultural status, socio-economic status, housing, income, occupation/profession; **position in immediate social and physical context:** housing, income, partnership and marriage, position in family, social networks (formal and informal); **personal history and biography:** (major) life events in general -being injured/personal trauma or disease-change in financial situation-change in living condition-death of family member/partner-divorce-getting married/starting to live together-life course, life events related to education -finishing an education-leaving an education unfinished-starting an education, life events related to work -being fifed-changing jobs-being involved in a reorganisation-starting a job-stopping working-work accident; **general mental personal factors/psychological assets:** acceptance, attitude, being in balance, beliefs -health beliefs, coping/coping style, creativity, desires/wishes, expectations, experiences, feelings, knowledge, learning style, locus of control, love of learning, meaningfullness, motives, needs, perceptions -perceived health-perseived stress, percistence/perseverance, preferences, psychosocial carrying capacity, purpose in life, quality of life/well-being, resilience/sense of coherence, self-efficacy, self-esteem/self-respect, self-management, spirituality/giving meaning to/sense of purpose, talents, values, zest for life; **disease-related factors:** acceptance of illness/disease/disorder, adherence/compliance with therapy, attribution, comorbidity, coping with illness, illness behaviour, illness beliefs, illness cognitions, illness perceptions, knowledge about a specific disease/disorder, wishes with respect to end of life, work ability, being in balance; **life-style (habits):** day-night rhythm, dietary habits, hobbies, movement habits, relaxation behavior, safety habits, smoking habits, sunbathing habits, use of alcohol, use of drugs, use of medication, use of personal care products; **work-related personal factors:**  ambition, attitude -to return to work-to stay at work, commitment/attachment to organisation/company, cooperation to return to work, employability/work ability, engagement/flow, expectations to be able to work, history of sickness absence, intention for turnover (work), intention to return to work, job satisfaction, management style, needs related to work -need for absenteeism-need for work, occupation/profession, occupational style, personal meaning of work, punctuality, safety habits at work, treshold for absenteeism, treshold for return to work, work agility, work identity, work history/work experience, work motivation, (disclosure) | 1, 2, 3, 4, 5, 6, 7, 8 |
| Hoefsmit 2014 | Positive perception of the situation, coping skills | 5, 7 |
| Holla 2020 | Stage of life, boredom, suffering, knowledge, positive experiences,attitude, risk perception, multiple lifestyle problems, habits, self-efficacy, self-control, intrinsic motivation, health condition, (goal setting, action planning, monitoring; fatigue, function impairments, appetite) | 3, 4, 5, 7, 8 |
| Hollenweger 2012* | Sex, ethnicity, social background | 1 |
| Hsieh 2013 | Age, gender, quality of life, (body mass index) | 1, 8 |
| Huang 2015 | Age, quality of life, (BMI) | 1, 8 |
| Huang 2018 | Age, socioeconomic status, overall behavioral patterns | 1, 7 |
| Huang 2020 | Age, gender | 1 |
| Huber 2010* | Health-related quality of life | 8 |
| Huer 2016 | Lifestyle, experinces, social/educational/professional back, race, gender, age, (individual personality traits) | 1, 3, 7 |
| Hwang 2014 | Age, gender, ethnicity | 1 |
| Ilieva 2013 | Age, gender, Healt behavior, coping strategies, co-morbidity | 1, 7, 8 |
| Jaarsma 2014 | Fear of injuries, fun/relaxation, not comfortable in the presence of other athletes, acceptance disability, more independence, self confidence, health/physical fitness, too busy with other activities, being dependent of others to be able to exercise, learning new skills, dealing with disability and aid, (weight control, social contacts, not being able to exercise because of physical disability, strength, loose energy) | 4, 5, 7 |
| Jacob 2017 | Age, race, socioeconomic status | 1 |
| Jaiswal 2019 | Onset of impairment, nature of impairment, willingness to explain about disability condition, challenges associated and ask for help or assistance. | 3, 8 |
| Jellema 2016 | Anxiety/hesitation, activity beliefs, priorities and options, confidence | 4, 5, 7 |
| Jobst 2013* | Helplessness, personal wishes, coping strategies, dependencies | 4, 6, 7, 8 |
| Joseph 2018 | Age, gender, living alone, 1 fall the last 3 months, time since stroke, comorbidity, (body mass index, using a walking aid) | 1, 2, 3, 8 |
| Kamath 2016 | Gender, age, income, family structure, family | 1, 2 |
| Kang 2017* | Patient participated in treatment and wanted to return to society as soon as possible with her mind full of will | 5 |
| Kembhavi 2011 | Self-esteem, self-efficacy, health behaviour, coping strategies for pain, sense of coherence | 7 |
| Kennedy 2020 | Age, gender, perception of self and disability, disease severity | 1, 5, 8 |
| Kim 2016 | Age, gender, education, (hand dominance, type of cerebral palsy) | 1 |
| Kirchberger 2010* | Feelings of being a burden, positive attitude, strategies of coping with the injury, faith and optimism, being optimistic | 5, 7 |
| Kirschneck 2011 | Patients’ knowledge of disease and treatment, life style, or attitude to pain and therapy, attitudes and beliefs about back pain, behaviours, coping and self-management, helplessness and independency, social status and job satisfaction | 1, 5, 7 |
| Kleffelgaard 2017 | Age, gender, level of education, post-injury sick-leave status, pre-injury employment, marital status, pre-injury comorbidities, cause of injury | 1, 2, 8 |
| Koehler 2011* | Lack of knowledge about osteoporosis, knowledge about disease, lack of information, dependence/independence, autonomy, nutritional habits, disease management, pain management, self-management, self-confidence, lifestyle, fitness level, agility, depression, comorbidity | 1, 5, 7, 8 |
| Kolehmainen 2011 | Age, emotions, child’s preferences, motivation, child’s confidence | 1, 4, 5, 7 |
| Kolehmainen 2015 | Age, emotion, beliefs about capabilities, motivation | 1, 4, 5, 7 |
| Kraft 2015 | **Feelings,** **cross-cutting, thoughts and beliefs, patterns of experience and behaviour, motives, activities and participation, feelings, (environmental):** helplessness, acceptanse, disease beliefs, pathways, agency Patterns of experience and behaviour, motives, activities and participation, or overall adjustment, worry, tension or apprehension, anger, poor mental health in general, reduced quality of life, disability acceptance, thoughts and beliefs including learned helplessness, self-efficacy, self-control, distorted representations of SCI-related disability, stress, fewer vocational interests and skills, sense of hope, patterns of experience and behaviour, alcohol and drug misuse, problem-solving abilities, motivational factors associations, selfreported depression, perceived stress levels, feelings of anxiety, difficulty coping with the challenges associated with one’s injury, (pain catastrophising) | 4, 5, 6, 7, 8 |
| Krauss 2017 | Motivation for physical excercise | 7 |
| Lakke 2013 | Age, gender, education, general health perceptions, vitality, mental health, body height, body weight | 1, 5, 7, 8 |
| Langer 2016 | Age, gender, disease severity | 1, 8 |
| Levo 2010 | Problems with uncertainty of life unpredictable course of disease, changes in lifestyle | 7, 8 |
| Lexell 2017 | A strong will and high self-esteem, a positive attitude, (high demands and a disciplined personality) | 5, 7 |
| Li 2015 | Acceptance of life in a wheelchair, acceptance of new sexuality, acceptance of new identity, perceived adjustment in familial roles, perceived adjustment in societal roles, knowledge deficit about spinal cord injury, adjustment to new body image, adaptation to new life style, coping with everyday life, coping with grief | 1, 7 |
| Li 2020 | Inferiority feelings caused by disabilities, knowledge about spinal cord injury, acceptance of life in a wheelchair/in bed,  coping with everyday life, adaptation to new life style, adjustment to new body image, adherence | 4, 5, 7 |
| Lilly 2019 | Lack of knowledge of adult healthcare system, lack self-efficacy, (family support) | 5, 7 |
| Lindsay 2016 | Body-image, motivation and self-efficacy, coping, planning and adaptation, independence, (identity, self-care) | 5, 7 |
| Liptak 2010 | Sex, age, race/ethnicity, socioeconomic status | 1 |
| Llewellyn 2018 | Depression, anxiety | 4, 8 |
| Manchaiah 2018 | Profession, past and current experience, overall behaviour patterns and characteristics, habits, coping styles, lifestyle | 1, 3, 7 |
| Manchaiah 2019 | Overall behaviour pattern and character style, coping styles, individual psychological assets | 7 |
| Manderoos 2018 | Age, height, self‐report health, self‐report fitness | 1, 7, 8 |
| Martins 2015* | Quality of life, self-efficacy, attitudes toward disability, formal education | 1, 5, 7, 8 |
| Marques 2014 | Sex, age, years of formal education, professional status, living arrangements, current marital status | 1, 2 |
| Meirte 2014 | Age, educational level, marital status, body image, coping style, (treatment regimes) | 1, 2, 5, 7 |
| Meirte 2016 | Age, educational level, marital status, body image, coping style, (treatment regimes) | 1, 2, 5, 7 |
| Menger 2017 | Importance, the impact of aphasia on emotional well-being | 5, 8 |
| Meyer 2016 | Age, gender, attitudes toward hearing devices, confidence in hearing device use, (family support, personality) | 1, 5 |
| Minis 2009 | Age, gender, education, type of occupation, marital status, expressed interest in employment, flexibility of employee, other diseases/comorbidities, quality of work, productivity of employee | 1, 2, 5, 7, 8 |
| Moorcroft 2019 | Age, gender, cultural background, attitude & behaviour, current health, (current abilities) | 1, 5, 7, 8 |
| Muijzer 2012 | Age, educational level, tenure, illness perception, attitude, self-efficacy, competencies | 1, 5, 7 |
| Muller 2014 | Socio-demographic factors, experiences and biography, emotional factors, satisfaction, behavioral and lifestyle factors, coping, motives/motivation, other health conditions, (social relationships, cognitive psychological factors, personality, biological/physiological factors) | 1 , 3, 4, 5, 7, 8 |
| Mulligan 2012 | Personal attributes: increasing age, unemployment, personal beliefs, lack of belief of interest in exercise, misunderstanding of what constitutes beneficial exercise, decreased self-efficacy for exercise, (lack of time, other responsibilities) | 1, 5, 7 |
| Murray 2017 | Needs, skills, comorbid disorders, (personality traits, attentional capacity) | 6, 7, 8 |
| Nam 2012* | Insight on prognosis, optimistic or pessimistic coping styles, behavioral patterns, suicidal ideas and attempts | 5, 7, 8 |
| Ng 2011* | **Demographic factors:** gender, race, age, educational status, socioeconomic status; **emotional states:** frustration, depression, stress, anxiety, fear, worry, degrading, grumpy, loss of confidence, anger, self-esteem, embarrassment; **hope:** both hopeful and hopeless, guilt, grief, loss, gratitude; **coping strategies and styles:** problem solving, search for information, planning, positivity, acceptance, humour, religion, using support, denial, avoidance; **personality:** stubborn, easy-going; **beliefs:** religious beliefs, self-esteem; **attitudes:** of the patient; **grateful attitude:** towards family and health professionals, fighting attitude, attitude towards assisted suicide, being organised, (“other” perceived support, personality stubborn, easy-going) | 1, 4, 5, 7 |
| Ng 2014 | Perceived fitness, physical activity intentions, global self-esteem | 5, 6, 7 |
| Nund 2015 | Attitude, acceptance of a new normal, remain hopeful | 5, 7 |
| Nuno 2018 | **Sociodemographic variables:** age, ethnicity, gender, level of education, marital status, occupational status, socioeconomic status; **habits and lifestyle:** dietary habits, drug use, fitness habits, lifestyle; **personality and other psychological characteristics:** agreeableness, attitudes towards health, autonomy, conscientiousness, extraversion, morality, motivation, neuroticism, openness to experience, optimism, personal attitude, resilience, self-awareness, self-esteem; **personal skills:** cognitive skills, coping skills, intelligence, psychosocial skills; **other personal factors: a**ge at onset, premorbid functioning, spirituality and religiosity, (personality, genetics) | 1, 2, 3, 7, 8 |
| Nuno 2019 | **Sociodemographic variables:** age, ethnicity, gender, level of education, living situation, marital status, occupational status, socioeconomic status; **habits and lifestyle:** drug use, lifestyle; **personality and other psychological characteristics:** agreeableness, attitudes towards health, autonomy, conscientiousness, extraversion, motivation, neuroticism, openness to experience, optimism, personal attitude, resilience, self-awareness, self-esteem, spirituality and religiosity; **personal skills**: cognitive skills, coping skills, intelligence, psychosocial skills; **other personal factors:** age at onset, genetics, personal history and biography, premorbid functioning, (personality) | 1 ,2 ,3 ,5 ,7 ,8 |
| O’Brien 2014 | Patient’s commitment to interventions, patient compliance to orthosis wear, arthritis grades had an important influence on successful prognosis, (therapists’ ability to communicate interventions in the learning style of the patient was important) | 8 |
| Offenbächer 2011 | Individual’s concurrent subjective experience, general patterns of behaviour, resilience , sense of coherence, mindfullness spirituality | 4, 7 |
| Oral 2013 | Self-efficacy, coping ability, persistence hard work and perseverance despite fatigue | 7 |
| Paanalahti 2013 | Coping style | 7 |
| Passier 2013 | Sex, age, education level, Income, marital status, passive coping, (neuroticism) | 1, 2, 7 |
| Pedras 2018 | Emotional reactions before and after lower limb amputation | 4 |
| Perenboom 2012 | Age, gender, perceived health, self-esteem, depressive symptoms | 1, 5, 7, 8 |
| Perfect 2020* | Epilepsia, confort issues, tremors, tiring, boredom, secondary medical condition, tired more quickly, (motivation) | 4, 5, 7, 8 |
| Pires 2018 | Frustration, anxiety, depression, negative sensory experience, loss of privacy, quality of life | 7, 8 |
| Pohl 2015 | Age, educational level, experienced falls, experienced fractures, perceived confidence of one’s balance, eating habits, other health conditions, comorbidities, emotional distress, depressive symptoms | 1, 3, 5, 7, 8 |
| Postma 2018 | Extraversion, agreeableness, conscientiousness, psychic stability, openness to experience, optimism, confidence, trustworthiness | 7, 8 |
| Pradat-Diehl 2012 | Social and psychosocial difficulties, previous and/or associated medical pathologies with functional implications,  behavioral disorders or associated psychiatric pathologies with functional implications | 3, 8 |
| Rauch 2013 | **Biographical, socio-demographical, and economical factors:** older/younger age, being female, working a lot/less/being without work/retired; **position in the immediate social and physical context:** not knowing/knowing other persons, being the only women, remoteness/closeness to services; **feelings (emotions and moods):** fear for health conditions and injuries/worsening of functioning level, feeling well after physical activities, fear/worry/respect/lack of joy/happiness, joy/fun/ liking in relation to physical activities, not feeling at ease with others/need for others/enjoying others in relation to physical activities, fear in the city and darkness/anger with environment/happiness with equipment/positive feelings in the nature, feeling unhappy/happy with services, changes in daily moods; **thoughts and beliefs (knowledge and concepts, evaluations, preferences, imaginations):** lack/possession of knowledge which type of physical activities is possible, lack of knowledge whom to contact regarding services/about range of services for physical activity, no benefit/comparison of physical activity before and after SCI/physical activity is not important or dangerous/benefit from physical activity/physical activity is important, being less capable than others/being a burden for others/having bad conscience toward others/being at the same level with others/being beneficial for others/having no bad conscience toward others, preferring other types of activities and participation than physical activity/preferring physical activity, imagination of performing types of physical activities; **motives (needs, interest and goals):** need for time for oneself when performing physical acitivity, need to sweat/release energy, no need to/need to be physically active, dependence on others/assistive devices/for outdoor activities, no interests/interest in general/in competitive/specific types of sport, no interest/interest in sport with others/with wheelchair-drivers/with able bodied persons, interest to perform physical activity in the nature, set goals to maintain health/to maintain/improve general health/mental health/physical health/fitness, to increase/maintain independence, to participate in competitions; **general patterns of experience and behavior (patterns/handling of thought, patterns/handling of motives, general behavior patterns):** lack of trust, inhibitions, shame/mental strength, positive thinking, self efficacy, repression/lack of acceptance/acceptance of disease, being lazy/homy/being ambitious, seeking mental and physical challenges, lack of strategies to overcome obstacles/successful coping strategies to perform physical activity, not doing things alone, not being a disciplined person/being a ‘movement’/‘competition-’/‘sportive-’/‘nature person’ | 1, 2, 5, 6, 7 |
| Ravesloot 2011 | Outcome expectations, sense of meaningfulness, self-efficacy, reinforcement management, (social norms) | 5, 6, 7 |
| Rekkedal 2017 | Sex, school grade, students’academic skills, motivation, self-confidence | 1, 7 |
| Renaud 2020 | Child sex, child age at injury, pre-injury behavioral functioning | 1, 3, 7 |
| Rio 2014 | Self area, emotivity, control, motivation, problem behaviors | 4, 7, 8 |
| Robinson 2011 | Overweight, obesity and extreme obesity, gender, age, nonhispanic white women, non-hispanic black women, income levels, smoked, underweight or normal weight, pregnant, coexisting health conditions | 1, 7, 8 |
| Rosa 2015 | Age, current occupation, marital status, (caregivers support) | 1, 2 |
| Rouquette2015 | Age, sex, (BMI, mental health) | 1 |
| Saal 2018 | Residents’ motivation to maintain mobility engage in social activities within their current living situation | 2, 7 |
| Saebu 2010 | Age, gender, unemployment, high intrinsic motivation, self efficacy, depression | 1, 7, 8 |
| Saebu 2011* | Age, gender, exercise self-schema, motivation for physical activity, perceived physical and mental health, time for activity | 1, 5, 7, 8 |
| Salvador-Carulla 2012 | Sociodemographics, major life events, family history, development history, past medical history, health related habits, psychological resistance to stress experiences, vitality, psychomatic reserve, (medical treatments, social reserve) | 1 ,3, 7 |
| Sánchez 2016 | Self-stigma, insight, disability acceptance, resilience, empathy, social competence | 5, 7 |
| Schmitt 2013 | Anxious: tense, uptight, irritable, difficulty in concentrating/relaxing, depressed: down-in-the-dumps, sad, in low spirits, pessimistic, unhappy, able to control: reduce/help your neck pain | 4, 7 |
| Schwegler2014 | Profession, education, family and marital status, social background, coping styles, lifestyle, overall behavior pattern and character style, individual psychological assets, past and current experience: past life events and concurrent events, other personal characteristics: e.g. personal expectations, beliefs and attitudes | 1, 2, 3, 5, 7 |
| Scott-Roberts 2018* | Planned adaptations | 7 |
| Seger 2017* | General personal characteristics: age, sex, attitudes, basic skills and behaviour patterns immediate life situation and socioeconomic/sociocultural factors, other health factors, (genetic factors, factors related to an individual’s physique, mental factors such as personality factors and cognitive and mnestic factors) | 1, 2, 5, 7, 8 |
| Segev 2019* | Age, military rank, previous employment experience, education acquired prior to and during the rehabilitation program, circumstances of the injury, type of military service: compulsory military service, permanent army service, the reserves | 1, 3, 8 |
| Sharma 2018 | Age, education, earnings, accounts, supplementary income, LTC insurance, currently married, family size, foreign-born, alcohol, smoking, depression, diabetes | 1, 2, 7, 8 |
| Simeonsson 2014* | Gender, race, age, education achieved/provided, profession, social background lived/provided, upbringing lived/provided, life experiences, past life events lived/provided, concurrent events lived/provided, individual psychological assets, fitness, lifestyle, habits, coping styles, overall behavior pattern, character style, other health conditions | 1, 3, 5, 7, 8 |
| Sivan 2011 | Patient/carer impressions, patient satisfaction | 5 |
| Sivan 2014* | Age, gender, experience, interest, purpose of life, motivation, independence, engagement, personal choice, convenience, competition, compliance, dominant side, (external look of device) | 1, 3, 5, 6, 7, 8 |
| Sivan 2016* | Personal view, purpose of life, personal goals, motivation, independence, confidence, frustration, comfort, tolerance,  compliance, (performance) | 5, 6, 7, 8 |
| Sjobbema 2018 | Age, education, financial difficulties, work history, previous experiences, fear of deteriorating health/capacity by RTW, job dissatisfaction, lack of knowledge/insight, lack of future expectations, low priority in work, irrational, perception of work capacity, perseption of illness, low self-efficacy, lack of motivation, illness behaviour, ineffective coping, unassertiveness, lack of receptivity, discontentment with medical treatment, non-compliance, secondary (financial) gain | 1, 3, 4, 5, 7, 8 |
| Smith 2016 | Finances, age, gender, race, level of education, marital status, years since injury, wheelchair factors – satisfaction,  loss of identity, alcohol use, tobacco use, physical health status, comorbidities, (personality) | 1, 2, 3, 5, 7, 8 |
| Soh 2020 | Age, gender, history of falls, depression, opioid use, bisphosphonates use, number of co-morbidities | 1, 3, 7, 8 |
| Stamm 2011 | Positive experience of the disease, being in control, being strong owing to having mastered the disease, positive attitude towards disease, struggle to master one’s life with the disease, such as unclear future, unclear outcome, needing to compromise, change of expectations, own attitudes towards SSc/being ill/accepting help/drugs, feeling guilt towards partners/family members/children, not viewing oneself as ill, focusing on what one can do, changing the way of doing things, adapting daily activities to own abilities, mental and physical exhaustion, impaired fitness as referring to the current individual state of the body, gender-specific differences: losing hair is terrible for a woman, change of gender roles in relationships | 5, 7, 8 |
| Stephens 2010 | Uncertainty of life, lifestyle changes | 7 |
| Stergiou-Kita 2014 | Age, gender, feelings of confidence, security and safety in the workplace, ability to establish job/ work goals, self-assessments of personal attributes/strengths and potential challenges return to work, expectations of recovery and perceptions of potential limitations, self-image/self-conciousness in relation to scarring, disfigurement, motivation/determination/perseverance | 1, 4, 7 |
| Sumathipala 2011 | Education, occupation, knowledge of health care services, attitudes towards health and ageing, striving for independence, reluctance to use public resources, comparison with other stroke survivors/ bereavement, health before the stroke/other health conditions | 1, 3, 5, 6 |
| Sung 2020 | Positive coping, self-efficacy, self-esteem | 7 |
| Suttiwong 2018 | Age, time since stroke, type of stroke, affected side | 1, 3, 8 |
| Tederko  2013 | Age, gender, education, health and legal awareness, expectations, athletic identity, life satisfaction, life purpose, lifestyle, coping strategies, (personality traits) | 1, 5, 6, 7 |
| Theis  2013 | Age, gender, race/ethnicity, education, employment status, marital status = environmental factor, retrieve health information from internet, current smoker, aerobic physical activity level | 1, 2, 5, 7 |
| Toovey  2019 | Age, fear associated with bike riding, child’s motivation/interest/own goal to ride a bike, motivation/interest to ride bike | 1, 4, 6 |
| Tschiesner 2010 | Socioeconomic status, smoking and alcohol use, individual coping strategies | 1, 7 |
| van Amelsvoort 2017 | (Sickness absence and work disability) |  |
| van Dijk  2017 | Age, satisfaction of the patient related to patient’s wishes and requirements’ | 1, 5 |
| van Gorp  2020 | Gender, age, nationality: Dutch or other, perceived self-competence, behavioural problems | 1, 7 |
| van Leeuwen 2016* | Demographic characteristics: e.g. age, sex, family situation: e.g. “patient has daughter of 3 years old”, personal attitudes of patients: e.g. “patient has a temperate attitude”, coping styles: e.g. “patient has difficulties in coping with HL”, health conditions and medical history: e.g. “high blood pressure”, “had many ear infection in her youth” | 1, 2, 5, 7, 8 |
| van Uem  2016 | Attitude, stress, education, ethnicity | 1, 5, 7 |
| van Wely  2020 | Gender: male/female, nationality Dutch/other, perceived competence, self-efficacy, coping style, (personality) | 1, 7 |
| Vander Werff 2016 | Gender, age, education, profession, social background, past and current experience, coping styles, overall behavior patterns | 1, 3, 7 |
| Vargus-Adams 2014 | Anxious about surgery, anxious, with oral aversion, eager to improve, goal to use her walker always at school with age, her behavior is more distinct from her peers and less acceptable in the classroom, poor safety awareness, very shy and introverted, chronic otitis media | 4, 5, 6, 7, 8 |
| Verboom  2011 | Age, gender, years of education, physical activity, chronic diseases, family history of MDD, (personality) | 1, 7, 8 |
| Verma  2020 | Negative thoughts, self-guilt, affected body image, and self-appearance, (no family support) | 4, 5 |
| Vieira  2011 | Language barriers, fear of falling, lack of knowledge by staff, lack of precautions, careless staff, lack of patient supervision, lack of awareness or by patients lack of awareness, increase staff awareness, increase patients’ awareness, patients’ choice and behaviour: improper footwear, lack of cooperation, not ringing the bell/asking for help, not waiting for help, not using walking devices, will for independence, (more patient supervision, more cooperation between staff, better communication, learn more from physiotherapists, proper footwear, establish regulations/guidelines, safety practices, education and behaviour) | 1, 4, 5, 7 |
| Virues-Ortega 2011 | Age, gender, education level, social status, (confidant, municipality, social contacts (amount), access to social and health resources) | 1 |
| Vooijs  2015 | Age, gender, educational level, race, substance use, use of medication and nocturnal toilet | 1, 7 |
| Wahlgren  2012 | Frustration with symptoms per DHII findings, avoidance of activities due to provocation of symptoms | 4, 7 |
| Wang  2013 | Gender, age, marital status | 1, 2 |
| Wang  2014 | Not feeling like a sense of burden to, depression, fear inadequately at work, emotional acceptance of disability, value and meaning of work, realistic in vocational goal, social dysfunctioning, (family/friend support) | 4, 5, 6, 8 |
| Warnink-Kavelaars  2019 | Selfesteem, self-competence, behavior | 5, 7 |
| Willis  2018 | Positive feelings associated with achieving goals, fun improved mood, emotional state , emotional release, conscious awareness (mindfulness), boredom, perception of benefits of activity, body image, increased insight, attitude, changes of attitude toward activity, improved attitude toward physical activity, increased awareness of own physical activity, change of habits, self-management, time management, lifestyle changes, motivation, independence, increased confidence, (improvement of entire family's attitude toward activity goal setting, emotional support) | 4, ,5, 7 |
| Worrall  2017* | Mood, successfully living with aphasia, severity of aphasia | 4, 7, 8 |
| Yen  2014 | Age, gender, education, marital status, previous fall | 1, 2, 8 |
| Zhu  2019 | Age, gender, education level, marriage status, time before treatment, comorbidities, hemorrhage type, whether to receive surgical treatment | 1, 2, 3, 8 |

*Study that was assessed as direct and highly relevant (level 6)

**References of included studies**

(1) Abeysekara P, Turchi R, O'Neil M. Obesity and children with special healthcare needs: special considerations for a special population. Curr Opin Pediatr (2014) 26:508-515. doi: 10.1097/MOP.0000000000000124

(2) Alcantara MA, De Souza RA, De Oliveira FA, Pinhal KC. Using the ICF framework to evaluate the effects of environmental factors on physical disability among people with diabetes mellitus. Physiother Theory Pract (2018) 31:1-8. doi: 10.1080/09593985.2018.1488191

(3) Amano T, Morrow-Howell N, Park S. Patterns of social engagement among older adults with mild cognitive impairment. J Gerontol B Psychol Sci Soc Sci (2020) 75:1361-1371. doi: 10.1093/geronb/gbz051

(4) Amatya B, Khan F, Galea MP. Optimizing post-acute care in breast cancer survivors: a rehabilitation perspective. J Multidiscip Healthc (2017) 30:347-357. doi: 10.2147/JMDH.S117362

(5) Amsters D, Schuurs S, Pershouse K, Power B, Harestad Y, Kendall M, et al. Factors Which Facilitate or Impede Interpersonal Interactions and Relationships after Spinal Cord Injury: A Scoping Review with Suggestions for Rehabilitation. Rehabil Res Pract (2016) 9373786: 1-13. doi: 10.1155/2016/9373786

(6) Andelic N, Stevens LF, Sigurdardottir S, Arango-Lasprilla JC, Roe C. Associations between disability and employment 1 year after traumatic brain injury in a working age population. Brain Inj (2012) 26:261-269. doi: 10.3109/02699052.2012.654589

(7) Andresen EM, Fried-Oken M, Peters B, Patrick DL. Initial constructs for patient-centered outcome measures to evaluate brain–computer interfaces. Disabil Rehabil Assist Technol (2016) 11:548-557. doi: 10.3109/17483107.2015.1027298

(8) Arnadottir SA, Gunnarsdottir ED, Stenlund H, Lundin-Olsson L. Determinants of self-rated health in old age: a population-based, cross-sectional study using the International Classification of Functioning. BMC Public Health (2011)11:670. doi: 10.1186/1471-2458-11-670

(9) Arnold CM, Gyurcsik NC. Risk factors for falls in older adults with lower extremity arthritis: a conceptual framework of current knowledge and future directions. Physiother Can (2012) 64:302-314. doi: 10.3138/ptc.2011-12BH

(10) Awad H, Alghadir A. Validation of the comprehensive international classification of functioning, disability and health core set for diabetes mellitus: physical therapists' perspectives. Am J Phys Med Rehabil (2013) 92:968-979. doi: 10.1097/PHM.0b013e31829b4a6d

(11) Ayvat F, Ayvat E, Kilinç Ö, Kilinç M, Yildirim SA. The International Classification of Functioning, Disability and Health-based factors related to physical activity level in adults with muscle diseases. Int J Rehabil Res (2019) 42:180-186. doi: 10.1097/MRR.0000000000000343

(12) Baert I, Vanlandewijck Y, Feys H, Vanhees L, Beyens H, Daly D. Determinants of cardiorespiratory fitness at 3, 6 and 12 months poststroke. Disabil Rehabil (2012) 34:1835-1842. doi: 10.3109/09638288.2012.665130

(13) Bagraith KS, Strong J. The International Classification of Functioning, Disability and Health (ICF) can be used to describe multidisciplinary clinical assessments of people with chronic musculoskeletal conditions. Clin Rheumatol (2013) 32:383-389. doi: 10.1007/s10067-012-2130-1

(14) Baird MW, Vargus-Adams J. Outcome measures used in studies of botulinum toxin in childhood cerebral palsy: a systematic review. J Child Neurol (2010) 25:721-727. doi: 10.1177/0883073809346846

(15) Barclay L, McDonald R, Lentin P. Social and community participation following spinal cord injury: a critical review. Int J Rehabil Res (2015) 38:1-19. doi: 10.1097/MRR.0000000000000085

(16) Batten H, Lamont R, Kuys S, McPhail S, Mandrusiak A. What are the barriers and enablers that people with a lower limb amputation experience when walking in the community? Disabil Rehabil (2020) 42: 3481-3487. doi: 10.1080/09638288.2019.1597177

(17) Bayly JL, Lloyd-Williams M. Identifying functional impairment and rehabilitation needs in patients newly diagnosed with inoperable lung cancer: a structured literature review. Support Care Cancer (2016) 24:2359-2379. doi: 10.1007/s00520-015-3066-1

(18) Beaudry L, Fortin S, Rochette A. Adapted dance used in subacute rehabilitation post-stroke: impacts perceived by patients, relatives and rehabilitation therapists. Disabil Rehabil (2019) 23:1-10. doi: 10.1080/09638288.2019.1581845

(19) Becker S, Kirchberger I, Cieza A, Berghaus A, Harréus U, Reichel O, et al. Content validation of the Comprehensive ICF Core Set for Head and Neck Cancer (HNC): the perspective of psychologists. Psycho Oncol (2010) 19:594-605. doi: 10.1002/pon.1608

(20) Berzina G, Paanalahti M, Lundgren-Nilsson Å, Sunnerhagen KS. Exploration of some personal factors with the International Classification of Functioning, Disability and Health core sets for stroke. J Rehabil Med (2013) 45:609-615. doi: 10.2340/16501977-1171

(21) Bilgin S, Cetin H, Karakaya J, Kose N. Multivariate Analysis of Risk Factors Predisposing to Kinesiophobia in Persons With Chronic Low Back and Neck Pain. J Manipulative Physiol Ther (2019) 42:565-571. doi: 10.1016/j.jmpt.2019.02.009

(22) Blake Huer M, Threats TT. Shared Responsibilities for Full Participation in Society: Planning Further Integration of the ICF Into AAC. Perspectives of the ASHA Special Interest Groups (2016) 1:83-93. 10.1044/persp1.SIG12.83

(23) Blake HL, McLeod S. Speech-language pathologists' support for multilingual speakers' English intelligibility and participation informed by the ICF. J Commun Disord (2019) 77:56-70. doi: 10.1016/j.jcomdis.2018.12.003

(24) Bogart KR, Lund EM, Rottenstein A. Disability pride protects self-esteem through the rejection-identification model. Rehabil Psychol (2018) 63:155-159. doi: 10.1037/rep0000166

(25) Bogart KR, Rottenstein A, Lund EM, Bouchard L. Who self-identifies as disabled? An examination of impairment and contextual predictors. Rehabil Psychol (2017) 62:553-562. doi: 10.1037/rep0000132

(26) Boldt C, Velstra IM, Brach M, Linseisen E, Cieza A. Nurses' intervention goal categories for persons with spinal cord injury based on the International Classification of Functioning, Disability and Health: an international Delphi survey. J Adv Nurs (2013) 69:1109-1124. doi: 10.1111/j.1365-2648.2012.06100.x.

(27) Boogaard S, De Vet HC, Faber CG, Zuurmond WW, Perez RS. An overview of predictors for persistent neuropathic pain. Expert Rev Neurother (2013) 13:505-513. doi: 10.1586/ern.13.44

(28) Boonen A, Maksymowych WP. Measurement: function and mobility (focussing on the ICF framework). Best Pract Res Clin Rheumatol (2010) 24:605-624. doi: 10.1016/j.berh.2010.05.008

(29) Bornbaum CC, Doyle PC, Skarakis-Doyle E, Theurer JA. A critical exploration of the International Classification of Functioning, Disability, and Health (ICF) framework from the perspective of oncology: recommendations for revision. J Multidiscip Healthc (2013) 6:75-86. doi: 10.2147/JMDH.S40020

(30) Bossmann T, Kirchberger I, Glaessel A, Stucki G, Cieza A. Validation of the Comprehensive ICF Core Set for Osteoarthritis: the perspective of physical therapists. Physiotherapy (2011) 97:3-16. doi: 10.1016/j.physio.2009.11.011

(31) Bours MJ, van der Linden, B. W., Winkels RM, van Duijnhoven FJ, Mols F, van Roekel EH, et al. Candidate Predictors of Health-Related Quality of Life of Colorectal Cancer Survivors: A Systematic Review. Oncologist (2016) 21:433-452. doi: 10.1634/theoncologist.2015-0258

(32) Brandt C, Janse van Vuuren, E. C. Dysfunction, activity limitations, participation restriction and contextual factors in South African women with pelvic organ prolapse. S Afr J Physiother (2019) 75:933. doi: 10.4102/sajp.v75i1.933

(33) Britto, H. M. J. S., Oliveira BS, Gomes CS, Pinto JM, Guerra RO. Contextual factors associated with life-space mobility in community-dwelling older adults based on International Classification of Functioning, Disability and Health: protocol for a systematic review. BMJ Open (2018) 8:e023468-023468. doi: 10.1136/bmjopen-2018-023468

(34) Bruls VEJ, Jansen NWH, van Kuijk, S. M. J., Kant I, Bastiaenen CHG. The course of complaints of arm, neck and/or shoulder: a cohort study in a university population participating in work or study. BMC Musculoskelet Disord (2018) 19:208-5. doi: 10.1186/s12891-018-2116-5

(35) Brütt AL, Magaard JL, Andreas S, Schulz H. A qualitative investigation of barriers and facilitators of rehabilitation success from the psychosomatic inpatients' perspective. Patient Prefer Adherence (2016)10:1881-1888. doi: 10.2147/PPA.S108117

(36) Burns AS, St-Germain D, Connolly M, Delparte JJ, Guindon A, Hitzig SL, et al. Phenomenological study of neurogenic bowel from the perspective of individuals living with spinal cord injury. Arch Phys Med Rehabil (2015) 96:49-55. doi: 10.1016/j.apmr.2014.07.417

(37) Chan F, Chia-Chiang Wang, Fitzgerald S, Muller V, Ditchman N, Menz F. Personal, environmental, and service-delivery determinants of employment quality for state vocational rehabilitation consumers: A multilevel analysis. J Vocat Rehabil (2016) 45:5-18. doi: 10.3233/JVR-160806

(38) Feng-Hang Chang (2013) Community participation among people who are homeless. [dissertation]. [Boston]: Boston University

(39) Chase JD, Lozano A, Hanlon A, Bowles KH. Identifying Factors Associated With Mobility Decline Among Hospitalized Older Adults. Clin Nurs Res (2018) 27:81-104. doi: 10.1177/1054773816677063

(40) Chiu TY, Yen CF, Escorpizo R, Chi WC, Liou TH, Liao HF, et al. What is the gap in activity and participation between people with disability and the general population in Taiwan? Int J Equity Health (2017) 16:136-5. doi: 10.1186/s12939-017-0628-5

(41) Chiu YJ, Boomer KB, Conyers LM. The Impact of Psychosocial Factors on Health and Retention Outcomes for People Living With HIV: Implications for Rehabilitation Counselors and Educators. Rehabil Couns Bull (2019) 62:94-107. doi: 10.1177/0034355218755304

(42) Choukou MA, Best KL, Potvin-Gilbert M, Routhier F, Lettre J, Gamache S, et al. Scoping review of propelling aids for manual wheelchairs. Assist Technol (2019) 28:1-15. doi: 10.1080/10400435.2019.1595789

(43) Cimarolli VR, Boerner K, Reinhardt JP, Horowitz A, Wahl HW, Schilling O, et al. A population study of correlates of social participation in older adults with age-related vision loss. Clin Rehabil (2017) 31:115-125. doi: 10.1177/0269215515624479

(44) Congdon WV, Ghazinouri R, Doshi S, Sykes C, Abraham J. Application of the ICF model in the acute care physical therapy management of a young man with pelvic chondrosarcoma following internal hemipelvectomy: a case report. Rehabil Oncol (2010) 28:3-9.

(45) Cornelius LR, van der Klink, J. J. L., Groothoff JW, Brouwer S. Prognostic Factors of Long Term Disability Due to Mental Disorders: A Systematic Review. J Occup Rehabil (2011) 21:259-274. doi: 10.1007/s10926-010-9261-5

(46) Covington KR, Atler KE, Schmid AA, Pergolotti M. Understanding fall risk for older adults with cancer: An evaluation of experts' perceptions. J Geriatr Oncol (2020) 11:263-269. doi: 10.1016/j.jgo.2019.06.006

(47) Culler KH, Wang YC, Byers K, Trierweiler R. Barriers and facilitators of return to work for individuals with strokes: perspectives of the stroke survivor, vocational specialist, and employer. Top Stroke Rehabil (2011) 18:325-340. doi: 10.1310/tsr1804-325

(48) de Beer J, Engels J, Heerkens Y, van der Klink J. Factors influencing work participation of adults with developmental dyslexia: a systematic review. BMC Public Health (2014) 24:77-77. doi: 10.1186/1471-2458-14-77

(49) de Kloet AJ, Gijzen R, Braga LW, Meesters JJL, Schoones JW, Vliet Vlieland, T. P. M. Determinants of participation of youth with acquired brain injury: A systematic review. Brain Inj (2015) 29:1135-1145. doi: 10.3109/02699052.2015.1034178

(50) de Rooij, I. J. M., van de Port, I. G. L., van der Heijden, L. L. M., Meijer JG, Visser-Meily JMA. Perceived barriers and facilitators for gait-related participation in people after stroke: From a patients' perspective. Physiother Theory Pract (2019) 3:1-9. doi: 10.1080/09593985.2019.1698085

(51) de Schipper E, Mahdi S, de Vries P, Granlund M, Holtmann M, Karande S, et al. Functioning and disability in autism spectrum disorder: A worldwide survey of experts. Autism Res (2016) 9:959-969. doi: 10.1002/aur.1592

(52) DeBoer BV, von der Luft G. Assessment of self-concept, self-esteem and self-worth in children with health conditions. Int J Child Adolesc Health (2010) 3:341-351.

(53) Delle Fave A, Bassi M, Allegri B, Cilia S, Falautano M, Goretti B, et al. Beyond Disease: Happiness, Goals, and Meanings among Persons with Multiple Sclerosis and Their Caregivers. Front Psychol (2017) 8:2216. doi: 10.3389/fpsyg.2017.02216

(54) Dempsey L, Skarakis-Doyle E. Developmental language impairment through the lens of the ICF: an integrated account of children's functioning. J Commun Disord (2010) 43:424-437. doi: 10.1016/j.jcomdis.2010.05.004

(55) den Ouden ME, Schuurmans MJ, Mueller-Schotte S, Brand JS, van der Schouw, Y. T. Domains contributing to disability in activities of daily living. J Am Med Dir Assoc (2013) 14:18-24. doi: 10.1016/j.jamda.2012.08.014

(56) Denver BD, Adolfsson M, Froude E, Rosenbaum P, Imms C, Deramore Denver B. Methods for conceptualising 'visual ability' as a measurable construct in children with cerebral palsy. BMC Med Res Methodol (2017) 17:1-13. doi: 10.1186/s12874-017-0316-6

(57) Ditchman N, Sheehan L, Rafajko S, Haak C, Kazukauskas K. Predictors of social integration for individuals with brain injury: An application of the ICF model. Brain Inj (2016) 30:1581-1589. doi: 10.1080/02699052.2016.1199900

(58) Dorstyn D, Black R, Mpofu E, Kneebone I. Utilizing the ICF to understand depressive symptomology in multiple sclerosis: An exploratory systematic review. Rehabil Psychol (2017) 62:143-164. doi: 10.1037/rep0000125

(59) Dutra, Fabiana C. M. S., Mancini MC, Neves JA, Kirkwood RN, Sampaio RF. Empirical analysis of the International Classification of Functioning, Disability and Health (ICF) using structural equation modeling. Rev Brasil Fisioter (2016) 20:384-394. doi: 10.1590/bjpt-rbf.2014.0168

(60) Dwyer KJ, Mulligan H. Community reintegration following spinal cord injury: Insights for health professionals in community rehabilitation services in New Zealand. Nz J Physiother (2015) 43:75-85. doi: 10.15619/NZJP/43.3.02

(61) Dür M, Coenen M, Stoffer MA, Fialka-Moser V, Kautzky-Willer A, Kjeken I, et al. Do patient-reported outcome measures cover personal factors important to people with rheumatoid arthritis? A mixed methods design using the International Classification of Functioning, Disability and Health as frame of reference. Health Qual Life Outcomes (2015) 13:27-8. doi: 10.1186/s12955-015-0214-8

(62) Earde PT, Praipruk A, Rodpradit P, Seanjumla P. Facilitators and Barriers to Performing Activities and Participation in Children With Cerebral Palsy: Caregivers' Perspective. Pediatr Phys Ther (2018) 30:27-32. doi: 10.1097/PEP.0000000000000459

(63) Ellis T, Cavanaugh JT, Earhart GM, Ford MP, Foreman KB, Fredman L, et al. Factors associated with exercise behavior in people with Parkinson disease. Phys Ther (2011) 91:1838-1848. doi: 10.2522/ptj.20100390

(64) Escorpizo R, Davis K, Stumbo T. Mapping of a standard documentation template to the ICF core sets for arthritis and low back pain. Physiother Res Int (2010) 15:222-231. doi: 10.1002/pri.466

(65) Esmail A, Poncet F, Auger C, Rochette A, Dahan-Oliel N, Labbé D, et al. The role of clothing on participation of persons with a physical disability: A scoping review. Appl Ergon (2020) 85:103058. doi: 10.1016/j.apergo.2020.103058

(66) European Physical and Rehabilitation Medicine Bodies Alliance. White Book on Physical and Rehabilitation Medicine (PRM) in Europe. Chapter 1. Definitions and concepts of PRM. Eur J Phys Rehabil Med (2018) 54:156-165. doi: 10.23736/S1973-9087.18.05144-4

(67) Fannin DK. The Intersection of Culture and ICF-CY Personal and Environmental Factors for Alternative and Augmentative Communication. Perspectives of the ASHA Special Interest Groups (2016) 1:63-82. doi: 10.1044/persp1.SIG12.63

(68) Farber RS, Kern ML, Brusilovsky E. Integrating the ICF with positive psychology: Factors predicting role participation for mothers with multiple sclerosis. Rehabil Psychol (2015) 60:169-178. doi: 10.1037/rep0000023

(69) Fekete C, Rauch A. Correlates and determinants of physical activity in persons with spinal cord injury: A review using the International Classification of Functioning, Disability and Health as reference framework. Disabil Health J (2012) 5:140-150. doi: 10.1016/j.dhjo.2012.04.003

(70) Finger ME, Boonen A, Woodworth TG, Escorpizo R, Christensen R, Nielsen SM, et al. An OMERACT Initiative Toward Consensus to Identify and Characterize Candidate Contextual Factors: Report from the Contextual Factors Working Group. J Rheumatol (2017) 44:1734-1739. doi: 10.3899/jrheum.161200

(71) Finger M, de Bie R, Selb M, Escorpizo R. An examination of concepts in vocational rehabilitation that could not be linked to the ICF based on an analysis of secondary data. Work (2016) 53:775-792. doi: 10.3233/WOR-152251

(72) Francescutti C, Gongolo F, Simoncello A, Frattura L. Description of the person-environment interaction: methodological issues and empirical results of an Italian large-scale disability assessment study using an ICF-based protocol. BMC Public Health (2011) 11 Suppl 4:S11. doi: 10.1186/1471-2458-11-S4-S11

(73) Fulcher AN, Purcell A, Baker E, Munro N. Factors influencing speech and language outcomes of children with early identified severe/profound hearing loss: Clinician-identified facilitators and barriers. Int J Speech Lang Pathol (2015) 17:325-333. doi: 10.3109/17549507.2015.1032351

(74) Gailey R, Clemens S, Sorensen J, Kirk-Sanchez N, Gaunaurd I, Raya M, et al. Variables that Influence Basic Prosthetic Mobility in People With Non-Vascular Lower Limb Amputation. PM R (2020) 12:130-139. doi: 10.1002/pmrj.12223

(75) Gan SM, Tung LC, Yeh CH, Chang HY, Wang CH. The ICF-CY-based structural equation model of factors associated with participation in children with autism. Dev Neurorehabil (2014) 17:24-33. doi: 10.3109/17518423.2013.835357

(76) Garner AA, Oʼconnor BC, Narad ME, Tamm L, Simon J, Epstein JN. The relationship between ADHD symptom dimensions, clinical correlates, and functional impairments. J Dev Behav Pediatr (2013) 34:469-477. doi: 10.1097/DBP.0b013e3182a39890

(77) Gass S, Kuhn M, Koenig I, Radlinger L, Koehler B. Development of an ICF-based questionnaire for urinary and/or fecal incontinence (ICF-IAF): The female patients' perspective using focus groups (subproject). Neurourol Urodyn (2019) 38:1657-1662. doi: 10.1002/nau.24031

(78) Geidl W, Semrau J, Pfeifer K. Health behaviour change theories: contributions to an ICF-based behavioural exercise therapy for individuals with chronic diseases. Disabil Rehabil (2014) 36:2091-2100. doi: 10.3109/09638288.2014.891056

(79) Geyh S, Peter C, Müller R, Stucki G, Cieza A. Translating topics in sci psychology into the international classification of functioning, disability and health. Top Spinal Cord Inj Rehabil (2011) 16:104-130. doi: 10.1310/sci1603-104

(80) Ginis KA, Arbour-Nicitopoulos KP, Latimer-Cheung AE, Buchholz AC, Bray SR, Craven BC, et al. Predictors of leisure time physical activity among people with spinal cord injury. Ann Behav Med (2012) 44:104-118. doi: 10.1007/s12160-012-9370-9

(81) Glaessel A, Kirchberger I, Stucki G, Cieza A. Does the comprehensive international classification of functioning, disability and health (ICF) core set for breast cancer capture the problems in functioning treated by physiotherapists in women with breast cancer? Physiotherapy (2011) 97:33-46. doi: 10.1016/j.physio.2010.08.010

(82) Glocker C, Kirchberger I, Gläßel A, Fincziczki A, Stucki G, Cieza A. Content validity of the comprehensive international classification of functioning, disability and health (ICF) core set for low back pain from the perspective of physicians: a Delphi survey. Chronic Illn (2013) 9:57-72. doi: 10.1177/1742395312451280

(83) Glässel A, Finger ME, Cieza A, Treitler C, Coenen M, Escorpizo R. Vocational rehabilitation from the client's perspective using the International Classification of Functioning, Disability and Health (ICF) as a reference. J Occup Rehabil (2011) 21:167-178. doi: 10.1007/s10926-010-9277-x

(84) Glässel A, Kirchberger I, Kollerits B, Amann E, Cieza A. Content Validity of the Extended ICF Core Set for Stroke: An International Delphi Survey of Physical Therapists. Phys Ther (2011) 91:1211-1222. doi: 10.2522/ptj.20100262

(85) Gomes DC, Longo E, de Camargo OK, de Sousa Dantas D, Ferreira HN, Regalado IC, et al. Common content between quality of life questionnaires for children with cystic fibrosis and the International Classification of Functionality, Disability and Health. J Rehabil Med (2019) 51:582-586. doi: 10.2340/16501977-2571

(86) Gradinger F, Köhler B, Khatami R, Mathis J, Cieza A, Bassetti C. Problems in functioning from the patient perspective using the International Classification of Functioning, Disability and Health (ICF) as a reference. J Sleep Res (2011) 20:171-182. doi: 10.1111/j.1365-2869.2010.00862.x

(87) Grotkamp S, Cibis W, Brüggemann S, Coenen MM, Gmünder HP, Keller K, et al. Personal Factors of the Bio-Psycho-Social Model (WHO): A Revised Classification by the German Society for Social Medicine and Prevention (DGSMP). Gesundheitswesen (2020) 82:107-116. doi: 10.1055/a-1011-3161

(88) Grunt S, Fieggen AG, Vermeulen RJ, Becher JG, Langerak NG. Selection criteria for selective dorsal rhizotomy in children with spastic cerebral palsy: a systematic review of the literature. Dev Med Child Neurol (2014) 56:302-312. doi: 10.1111/dmcn.12277

(89) Hamed R, Tariah HA, Hawamdeh ZM. Personal Factors Affecting the Daily Functioning and Well-Being of Patients with Multiple Sclerosis Using the International Classification of Functioning Model. Int J Ment Health (2012) 41:47-61. doi: 10.2753/IMH0020-7411410404

(90) Hancock AB. An ICF Perspective on Voice-related Quality of Life of American Transgender Women. J Voice (2017) 31:115.e1-115.e8. doi: 10.1016/j.jvoice.2016.03.013

(91) Hand, BN. (2016) Caregiver Burden, Participation, and Sensory Subtypes in Children with Autism. [dissertation]. [Ohio]: The Ohio State University

(92) Hansen AØ, Kristensen HK, Cederlund R, Tromborg H. Test-retest reliability of Antonovsky's 13-item sense of coherence scale in patients with hand-related disorders. Disabil Rehabil (2017) 39:2105-2111. doi: 10.1080/09638288.2016.1215555

(93) Hardy TLD, Boliek CA, Wells K, Rieger JM. The ICF and Male-to-Female Transsexual Communication. Int J Transgender (2013) 14:196-208. doi: 10.1080/15532739.2014.890561

(94) Hassett LM, Tate RL, Moseley AM, Gillett LE. Injury severity, age and pre-injury exercise history predict adherence to a home-based exercise programme in adults with traumatic brain injury. Brain Inj (2011) 25:698-706. doi: 10.3109/02699052.2011.579934

(95) Hawkins BL, McGuire FA, Britt TW, Linder SM. Identifying contextual influences of community reintegration among injured servicemembers. J Rehabil Res Dev (2015) 52:235-246. doi: 10.1682/JRRD.2014.08.0195

(96) Heerkens YF, de Brouwer, C. P. M., Engels JA, van der Gulden, J. W. J., Kant I. Elaboration of the contextual factors of the ICF for Occupational Health Care. Work (2017) 57:187-204. doi: 10.3233/WOR-172546

(97) Hoefsmit N, Houkes I, Nijhuis F. Environmental and personal factors that support early return-to-work: a qualitative study using the ICF as a framework. Work (2014) 48:203-215. doi: 10.3233/WOR-131657

(98) Holla JFM, van den Akker, L. E., Dadema T, de Groot S, Tieland M, Weijs PJM, et al. Determinants of dietary behaviour in wheelchair users with spinal cord injury or lower limb amputation: Perspectives of rehabilitation professionals and wheelchair users. PLoS One (2020) 15:e0228465. doi: 10.1371/journal.pone.0228465

(99) Hollenweger J, Moretti M. Using the International Classification of Functioning, Disability and Health Children and Youth version in education systems: a new approach to eligibility. Am J Phys Med Rehabil (2012) 91:97-102. doi: 10.1097/PHM.0b013e31823d5501

(100) Hsieh RL, Lee WC, Lo MT, Liao WC. Postural stability in patients with knee osteoarthritis: comparison with controls and evaluation of relationships between postural stability scores and International Classification of Functioning, Disability and Health components. Arch Phys Med Rehabil (2013) 94:340-346. doi: 10.1016/j.apmr.2012.09.022

(101) Huang HH. Perspectives on Early Power Mobility Training, Motivation, and Social Participation in Young Children with Motor Disabilities. Front Psychol (2018) 8:2330. doi: 10.3389/fpsyg.2017.02330

(102) Huang KH, Hsieh RL, Lee WC. Pain, Physical Function, and Health in Patients With Knee Osteoarthritis. Rehabil Nurs (2017) 42:235-241. doi: 10.1002/rnj.234

(103) Huang SW, Lin LF, Chang KH, Escorpizo R, Liou TH. Development of a comprehensive core set from the international classification of functioning, disability and health for return to work among patients with stroke through delphi-based consensus. Eur J Phys Rehabil Med (2020) 56: 257-264. doi: 10.23736/S1973-9087.20.05992-4

(104) Huber JG, Sillick J, Skarakis-Doyle E. Personal perception and personal factors: incorporating health-related quality of life into the International Classification of Functioning, Disability and Health. Disabil Rehabil (2010) 32:1955-1965. doi: 10.3109/09638281003797414

(105) Hwang AW, Liao HF, Chen PC, Hsieh WS, Simeonsson RJ, Weng LJ, et al. Applying the ICF-CY framework to examine biological and environmental factors in early childhood development. J Formos Med Assoc (2014) 113:303-312. doi: 10.1016/j.jfma.2011.10.004

(106) Ilieva EM, Oral A, Küçükdeveci AA, Varela E, Valero R, Berteanu M, et al. Osteoarthritis. The role of physical and rehabilitation medicine physicians. The European perspective based on the best evidence. A paper by the UEMS-PRM Section Professional Practice Committee. Eur J Phys Rehabil Med (2013) 49:579-593.

(107) Jaarsma EA, Geertzen JHB, Jong R, Dijkstra PU, Dekker R. Barriers and facilitators of sports in Dutch Paralympic athletes: An explorative study. Scand J Med Sci Sports (2014) 24:830-836. doi: 10.1111/sms.12071

(108) Jacob M, Cox SR. Examining transgender health through the International Classification of Functioning, Disability, and Health's (ICF) Contextual Factors. Qual Life Res (2017) 26:3177-3185. doi: 10.1007/s11136-017-1656-8

(109) Jaiswal A, Aldersey HM, Wittich W, Mirza M, Finlayson M. Using the ICF to Identify Contextual Factors That Influence Participation of Persons With Deafblindness. Arch Phys Med Rehabil (2019) 100:2324-2333. doi: 10.1016/j.apmr.2019.03.010

(110) Jellema S, van der Sande R, van Hees S, Zajec J, Steultjens EM, Nijhuis-van der Sanden, Maria W. Role of Environmental Factors on Resuming Valued Activities Poststroke: A Systematic Review of Qualitative and Quantitative Findings. Arch Phys Med Rehabil (2016) 97:991-1002.e1. doi: 10.1016/j.apmr.2016.01.015

(111) Jobst A, Kirchberger I, Cieza A, Stucki G, Stucki A. Content Validity of the Comprehensive ICF Core Set for Chronic Obstructive Pulmonary Diseases: An International Delphi Survey. Open Respir Med J (2013) 7:33-45. doi: 10.2174/1874306401307010033

(112) Joseph C, Conradsson D, Hagströmer M, Lawal I, Rhoda A. Objectively assessed physical activity and associated factors of sedentary behavior among survivors of stroke living in Cape Town, South Africa. Disabil Rehabil (2018) 40:2509-2515. doi: 10.1080/09638288.2017.1338761

(113) Kamath S, Fayed N, Goodman C, Streiner DL, Ronen GM. Extracurricular participation among children with epilepsy in Canada. Epilepsy Behav (2016) 56:118-122. doi: 10.1016/j.yebeh.2015.12.028

(114) Kang TW, Cynn HS. Progressive intervention strategy for the gait of sub-acute stroke patient using the International Classification of Functioning, Disability, and Health tool. NeuroRehabilitation (2017) 40:473-481. doi: 10.3233/NRE-171434

(115) Kembhavi G, Darrah J, Payne K, Plesuk D. Adults with a diagnosis of cerebral palsy: a mapping review of long-term outcomes. Dev Med Child Neurol (2011) 53:610-614. doi: 10.1111/j.1469-8749.2011.03914.x

(116) Kennedy RA, Carroll K, McGinley JL, Paterson KL. Walking and weakness in children: a narrative review of gait and functional ambulation in paediatric neuromuscular disease. J Foot Ankle Res (2020) 13:10. doi: 10.1186/s13047-020-0378-2

(117) Kim HY. An investigation of the factors affecting handwriting articulation of school aged children with cerebral palsy based on the international classification of functioning, disability and health. J Phys Ther Sci (2016) 28:347-350. doi: 10.1589/jpts.28.347

(118) Kirchberger I, Sinnott A, Charlifue S, Kovindha A, Lüthi H, Campbell R, et al. Functioning and disability in spinal cord injury from the consumer perspective: an international qualitative study using focus groups and the ICF. Spinal Cord (2010) 48:603-613. doi: 10.1038/sc.2009.184

(119) Kirschneck M, Kirchberger I, Amann E, Cieza A. Validation of the comprehensive ICF core set for low back pain: the perspective of physical therapists. Man Ther (2011) 16:364-372. doi: 10.1016/j.math.2010.12.011

(120) Kleffelgaard I, Langhammer B, Hellstrom T, Sandhaug M, Tamber AL, Soberg HL. Dizziness-related disability following mild–moderate traumatic brain injury. Brain Inj (2017) 31:1436-1444. doi: 10.1080/02699052.2017.1377348

(121) Koehler B, Kirchberger I, Glaessel A, Kool J, Stucki G, Cieza A. Validation of the International Classification of Functioning, Disability and Health Comprehensive Core Set for Osteoporosis: the perspective of physical therapists. J Geriatr Phys Ther (2011) 34:117-130. doi: 10.1519/JPT.0b013e31820aa990

(122) Kolehmainen N, Francis JJ, Ramsay CR, Owen C, McKee L, Ketelaar M, et al. Participation in physical play and leisure: developing a theory- and evidence-based intervention for children with motor impairments. BMC Pediatr (2011) 11:100-100. doi: 10.1186/1471-2431-11-100

(123) Kolehmainen N, Ramsay C, McKee L, Missiuna C, Owen C, Francis J. Participation in Physical Play and Leisure in Children With Motor Impairments: Mixed-Methods Study to Generate Evidence for Developing an Intervention. Phys Ther (2015) 95:1374-1386. doi: 10.2522/ptj.20140404

(124) Kraft R, Dorstyn D. Psychosocial correlates of depression following spinal injury: A systematic review. J Spinal Cord Med (2015) 38:571-583. doi: 10.1179/2045772314Y.0000000295

(125) Krauss I, Katzmarek U, Rieger MA, Sudeck G. Motives for physical exercise participation as a basis for the development of patient-oriented exercise interventions in osteoarthritis: a cross-sectional study. Eur J Phys Rehabil Med (2017) 53:590-602. doi: 10.23736/S1973-9087.17.04482-3

(126) Lakke SE, Soer R, Geertzen JH, Wittink H, Douma RK, van der Schans, C. P., et al. Construct validity of functional capacity tests in healthy workers. BMC Musculoskelet Disord (2013) 14:180. doi: 10.1186/1471-2474-14-180

(127) Langer D, Maeir A, Michailevich M, Applebaum Y, Luria S. Using the international classification of functioning to examine the impact of trigger finger. Disabil Rehabil (2016) 38:2530-2537. doi: 10.3109/09638288.2015.1137980

(128) Levo H, Stephens D, Poe D, Kentala E, Pyykkö I. Use of ICF in assessing the effects of Meniere's disorder on life. Ann Otol Rhinol Laryngol (2010)119:583-589. doi: 10.1177/000348941011900903

(129) Lexell EM, Langdell I, Lexell J. Vocational situation and experiences from the work environment among individuals with neuromuscular diseases. Work (2017) 56:519-530. doi: 10.3233/WOR-172527

(130) Li K, Xie S, Wang Y, Tang J, He X, Liu T, et al. Outcome indicators in the transitional care of people with spinal cord injury in China: a Delphi survey based on the International Classification of Functioning, Disability and Health. Disabil Rehabil (2020) 42:1539-1547. doi: 10.1080/09638288.2018.1528638

(131) Li K, Yan T, You L, Li R, Ross AM. International classification of functioning, disability and health categories for spinal cord injury nursing in China. Disabil Rehabil (2015) 37:25-32. doi: 10.3109/09638288.2014.890674

(132) Lilly H, Bitzel M, Pejnovic T, Schnell J, Doty A. Barriers and characteristics for successful transition to adult healthcare for individuals with cerebral palsy: a systematic review. Phys Ther Rev (2019) 24:195-207. doi: 10.1080/10833196.2019.1662995

(133) Lindsay S. Child and youth experiences and perspectives of cerebral palsy: a qualitative systematic review. Child Care Health Dev (2016) 42:153-175. doi: 10.1111/cch.12309

(134) Liptak GS, Kennedy JA, Dosa NP. Youth with spina bifida and transitions: health and social participation in a nationally represented sample. J Pediatr (2010) 157:584-8, 588.e1.doi: 10.1016/j.jpeds.2010.04.004

(135) Llewellyn A, McCabe CS, Hibberd Y, White P, Davies L, Marinus J, et al. Are you better? A multi-centre study of patient-defined recovery from Complex Regional Pain Syndrome. Eur J Pain (2018) 22:551-564. doi: 10.1002/ejp.1138

(136) Manchaiah V, Beukes EW, Granberg S, Durisala N, Baguley DM, Allen PM, et al. Problems and Life Effects Experienced by Tinnitus Research Study Volunteers: An Exploratory Study Using the ICF Classification. J Am Acad Audiol (2018) 29:936-947. doi: 10.3766/jaaa.17094

(137) Manchaiah V, Granberg S, Grover V, Saunders GH, Ann Hall D. Content validity and readability of patient-reported questionnaire instruments of hearing disability. Int J Audiol (2019) 58:565-575. doi: 10.1080/14992027.2019.1602738

(138) Manderoos S, Vaara M, Karppi SL, Aunola S, Puukka P, Surakka J, et al. Power of lower extremities is most important determinant of agility among physically inactive or active adult people. Physiother Res Int (2018) 23:e1716. doi: 10.1002/pri.1716

(139) Marques A, Martins A, Jácome C, Figueiredo D. Linking the EASY-care standard to the international classification of functioning, disability and health. Disabil Rehabil (2014) 36:593-599. doi: 10.3109/09638288.2013.804598

(140) Martins AC. Using the International Classification of Functioning, Disability and Health (ICF) to address facilitators and barriers to participation at work. Work (2015) 50:585-593. doi: 10.3233/WOR-141965

(141) Meirte J, van Loey, N. E. E., Maertens K, Moortgat P, Hubens G, Van Daele U. Classification of quality of life subscales within the ICF framework in burn research: Identifying overlaps and gaps. Burns (2014) 40:1353-1359. doi: 10.1016/j.burns.2014.01.015

(142) Meirte, J. (2016) The ICF as a Framework for Post Burn Dysfunctioning: Evaluation, Quality of Life and Vacuum massage in Patients with Hypertrophic Burn Scars. [dissertation] [Antwerp]: University of Antwerp

(143) Menger F, Morris J, Salis C. Internet Use in Aphasia. Top Lang Disord (2017) 37:6-24. doi: 10.1097/TLD.0000000000000110

(144) Meyer C, Grenness C, Scarinci N, Hickson L. What Is the International Classification of Functioning, Disability and Health and Why Is It Relevant to Audiology? Semin Hear (2016) 37:163-186. doi: 10.1055/s-0036-1584412

(145) Minis M, Heerkens Y, Engels J, Oostendorp R, van Engelen B. Classification of employment factors according to the International Classification of Functioning, Disability and Health in patients with neuromuscular diseases: a systematic review. Disabil Rehabil (2009) 31:2150-2163. doi: 10.3109/09638280902951838

(146) Moorcroft A, Scarinci N, Meyer C. A systematic review of the barriers and facilitators to the provision and use of low-tech and unaided AAC systems for people with complex communication needs and their families. Disabil Rehabil Assist Technol (2019) 14:710-731. doi: 10.1080/17483107.2018.1499135

(147) Muijzer A, Geertzen JH, de Boer WE, Groothoff JW, Brouwer S. Identifying factors relevant in the assessment of return-to-work efforts in employees on long-term sickness absence due to chronic low back pain: a focus group study. BMC Public Health (2012) 24:77. doi: 10.1186/1471-2458-12-77

(148) Mulligan HF, Hale LA, Whitehead L, Baxter GD. Barriers to physical activity for people with long-term neurological conditions: a review study. Adapt Phys Activ Q (2012) 29:243-265. doi: 10.1123/apaq.29.3.243

(149) Murray E, Iuzzini-Seigel J. Efficacious Treatment of Children With Childhood Apraxia of Speech According to the International Classification of Functioning, Disability and Health. Perspectives of the ASHA Special Interest Groups (2017) 2:61-76. doi: 0.1044/persp2.SIG2.61

(150) Müller R, Geyh S. Lessons learned from different approaches towards classifying personal factors. Disabil Rehabil (2015) 37:430-438. doi: 10.3109/09638288.2014.923527

(151) Nam HS, Kim KD, Shin HI. ICF Based Comprehensive Evaluation for Post-Acute Spinal Cord Injury. Ann Rehabil Med (2012) 36:804-814. doi: 10.5535/arm.2012.36.6.804

(152) Ng K, Välimaa R, Rintala P, Tynjälä J, Villberg J, Kannas L. Self-esteem and intentions mediate perceived fitness with physical activity in Finnish adolescents with long-term illness or disabilities. Acta Gymnica (2014) 44:185-192. doi:10.5507/ag.2014.019

(153) Ng L, Khan F. Identification of personal factors in motor neurone disease: a pilot study. Rehabil Res Pract (2011) 2011:871237. doi: 10.1155/2011/871237

(154) Nund RL, Scarinci NA, Cartmill B, Ward EC, Kuipers P, Porceddu SV. Third-party disability in carers of people with dysphagia following non-surgical management for head and neck cancer. Disabil Rehabil (2016) 38:462-471. doi: 10.3109/09638288.2015.1046563

(155) Nuño L, Barrios M, Rojo E, Gómez-Benito J, Guilera G. Validation of the ICF Core Sets for schizophrenia from the perspective of psychiatrists: An international Delphi study. J Psychiatr Res (2018) 103:134-141. doi: 10.1016/j.jpsychires.2018.05.012

(156) Nuño L, Guilera G, Coenen M, Rojo E, Gómez-Benito J, Barrios M. Functioning in schizophrenia from the perspective of psychologists: A worldwide study. PLoS One (2019) 14:e0217936. doi: 10.1371/journal.pone.0217936

(157) O'Brien VH, McGaha JL. Current practice patterns in conservative thumb CMC joint care: survey results. J Hand Ther (2014) 27:14-22. doi: 10.1016/j.jht.2013.09.001

(158) Offenbächer M, Sauer S, Hieblinger R, Hufford DJ, Walach H, Kohls N. Spirituality and the International Classification of Functioning, Disability and Health: content comparison of questionnaires measuring mindfulness based on the International Classification of Functioning. Disabil Rehabil (2011) 33:2434-2445. doi: 10.3109/09638288.2011.573902

(159) Oral A, Yaliman A. Revisiting the management of fatigue in multiple sclerosis in the context of rehabilitation: a narrative review of current evidence. Int J Rehabil Res (2013) 36:97-104. doi: 10.1097/MRR.0b013e32835fd9b2

(160) Paanalahti M, Lundgren-Nilsson A, Arndt A, Sunnerhagen KS. Applying the Comprehensive International Classification of Functioning, Disability and Health Core Sets for stroke framework to stroke survivors living in the community. J Rehabil Med (2013) 45:331-340. doi: 10.2340/16501977-1110

(161) Passier, P E C A., Visser-Meily J, Rinkel GJE, Lindeman E, Post MWM. Determinants of health-related quality of life after aneurysmal subarachnoid hemorrhage: a systematic review. Qual Life Res (2013) 22:1027-1043. doi: 10.1007/s11136-012-0236-1

(162) Pedras S, Carvalho R, Pereira MG. A predictive model of anxiety and depression symptoms after a lower limb amputation. Disabil Health J (2018) 11:79-85. doi: 10.1016/j.dhjo.2017.03.013

(163) Perenboom RJ, Wijlhuizen GJ, Garre FG, Heerkens YF, van Meeteren NL. An empirical exploration of the relations between the health components of the International Classification of Functioning, Disability and Health (ICF). Disabil Rehabil (2012) 34:1556-1561. doi: 10.3109/09638288.2011.647233

(164) Perfect E, Hoskin E, Noyek S, Davies TC. A systematic review investigating outcome measures and uptake barriers when children and youth with complex disabilities use eye gaze assistive technology. Dev Neurorehabil (2020) 23:145-159. doi: 10.1080/17518423.2019.1600066

(165) Pires JM, Ferreira AM, Rocha F, Andrade LG, Campos I, Margalho P, et al. Assessment of neurogenic bowel dysfunction impact after spinal cord injury using the International Classification of Functioning, Disability and Health. Eur J Phys Rehabil Med (2018) 54:873-879. doi: 10.23736/S1973-9087.18.04991-2

(166) Pohl P, Ahlgren C, Nordin E, Lundquist A, Lundin-Olsson L. Gender perspective on fear of falling using the classification of functioning as the model. Disabil Rehabil (2015) 37:214-222. doi: 10.3109/09638288.2014.914584

(167) Postma SAE, van Boven K, Ten Napel H, Gerritsen DL, Assendelft WJJ, Schers H, et al. The development of an ICF-based questionnaire for patients with chronic conditions in primary care. J Clin Epidemiol (2018) 103:92-100. doi: 10.1016/j.jclinepi.2018.07.005

(168) Pradat-Diehl P, Joseph P-, Beuret-Blanquart F, Luauté J, Tasseau F, Remy-Neris O, et al. Physical and rehabilitation medicine (PRM) care pathways: Adults with severe traumatic brain injury. Annals of Physical and Rehabilitation Medicine (2012) 55:546-556. doi: 10.1016/j.rehab.2012.07.002

(169) Rauch A, Fekete C, Cieza A, Geyh S, Meyer T. Participation in physical activity in persons with spinal cord injury: a comprehensive perspective and insights into gender differences. Disabil Health J (2013) 6:165-176. doi: 10.1016/j.dhjo.2013.01.006

(170) Ravesloot C, Ruggiero C, Ipsen C, Traci M, Seekins T, Boehm T, et al. Disability and health behavior change. Disabil Health J (2011) 4:19-23. doi: 10.1016/j.dhjo.2010.05.006

(171) Rekkedal AM. Factors associated with school participation among students with hearing loss. Scand J Disabil Res (2017) 19:175-193. doi: 10.1080/15017419.2016.1167771

(172) Renaud MI, Lambregts SAM, van de Port, I. G. L., Catsman-Berrevoets CE, van Heugten CM. Predictors of activities and participation six months after mild traumatic brain injury in children and adolescents. Eur J Paediatr Neurol (2020) 25: 145-156. doi: 10.1016/j.ejpn.2019.11.008

(173) Rio L, Damiani P, Paloma FG. Physical activities and special educational needs. J Hum Sport Exerc (2015) 10:S447-S454. doi: 10.14198/jhse.2015.10.Proc1.38

(174) Robinson KT, Butler J. Understanding the causal factors of obesity using the International Classification of Functioning, Disability and Health. Disabil Rehabil (2011) 33:643-651. doi: 10.3109/09638288.2010.505994

(175) Rosa MC, Marques A, Demain S, Metcalf CD. Knee posture during gait and global functioning post-stroke: a theoretical ICF framework using current measures in stroke rehabilitation. Disabil Rehabil (2015) 37:904-913. doi: 10.3109/09638288.2014.948132

(176) Rouquette A, Badley EM, Falissard B, Dub T, Leplege A, Coste J. Moderators, mediators, and bidirectional relationships in the International Classification of Functioning, Disability and Health (ICF) framework: An empirical investigation using a longitudinal design and Structural Equation Modeling (SEM). Soc Sci Med (2015) 135:133-142. doi: 10.1016/j.socscimed.2015.05.007

(177) Saal S, Meyer G, Beutner K, Klingshirn H, Strobl R, Grill E, et al. Development of a complex intervention to improve participation of nursing home residents with joint contractures: a mixed-method study. BMC Geriatr (2018) 18:61. doi: 10.1186/s12877-018-0745-z

(178) Saebu M, Sørensen M. Factors associated with physical activity among young adults with a disability. Scand J Med Sci Sports (2011) 21:730-738. doi: 10.1111/j.1600-0838.2010.01097.x

(179) Saebu M. Physical Disability and Physical Activity: a Review of the Literature on Correlates and Associations. Eur J of Adapt Phys Act (2010) 3:37-55. doi: 10.5507/euj.2010.008

(180) Salvador-Carulla L, Walsh CO, Alonso F, Gómez R, de Teresa C, Cabo-Soler JR, et al. eVITAL: a preliminary taxonomy and electronic toolkit of health-related habits and lifestyle. Sci World J (2012) 2012:379752. doi: 10.1100/2012/379752

(181) Schmitt MA, Schröder CD, Stenneberg MS, van Meeteren NL, Helders PJ, Pollard B, et al. Content validity of the Dutch version of the Neck Bournemouth Questionnaire. Man Ther (2013) 18:386-389. doi: 10.1016/j.math.2013.01.004

(182) Schwegler U, Anner J, Glässel A, Brach M, De Boer W, Cieza A, et al. Towards comprehensive and transparent reporting: context-specific additions to the ICF taxonomy for medical evaluations of work capacity involving claimants with chronic widespread pain and low back pain. BMC Health Serv Res (2014) 14:361. doi: 10.1186/1472-6963-14-361

(183) Scott-Roberts S, Purcell C. Understanding the Functional Mobility of Adults with Developmental Coordination Disorder (DCD) Through the International Classification of Functioning (ICF). Curr Dev Disord Rep (2018) 5:26-33. doi: 10.1007/s40474-018-0128-3

(184) Seger W, Grotkamp S, Cibis W. Personal factors and their relevance for the assessment and allocation of benefits in social medicine and rehabilitation. Electron Physician (2017) 9:5868-5870. doi: 10.19082/5868

(185) Segev D, Schiff M. Integrating Israeli Defense Force (IDF) veterans with disabilities into the workforce: characteristics and predictors. Isr J Health Policy Res (2019) 8:86-2. doi: 10.1186/s13584-019-0352-2

(186) Sharma A. Body mass index and mobility limitations: An analysis of middle-aged and older Black, Hispanic, and White women in the U.S. Obes Res Clin Pract (2018) 12:547-554. doi: 10.1016/j.orcp.2018.06.001

(187) Simeonsson RJ, Lollar D, Björck-Åkesson E, Granlund M, Brown SC, Zhuoying Q, et al. ICF and ICF-CY lessons learned: Pandora's box of personal factors. Disabil Rehabil (2014) 36:2187-2194. doi: 10.3109/09638288.2014.892638

(188) Sivan M, Gallagher J, Holt R, Weightman A, Levesley M, Bhakta B. Investigating the International Classification of Functioning, Disability, and Health (ICF) Framework to Capture User Needs in the Concept Stage of Rehabilitation Technology Development. Assist Technol (2014) 26:164-173. doi: 10.1080/10400435.2014.903315

(189) Sivan M, Gallagher J, Holt R, Weightman A, Levesley M, Bhakta B. Investigating the International Classification of Functioning, Disability, and Health (ICF) Framework to Capture User Needs in the Concept Stage of Rehabilitation Technology Development. Assist Technol (2014) 26:164-173. doi: 10.1080/10400435.2014.903315

(190) Sivan M, O'Connor RJ, Makower S, Levesley M, Bhakta B. Systematic review of outcome measures used in the evaluation of robot-assisted upper limb exercise in stroke. J Rehabil Med (2011) 43:181-189. doi: 10.2340/16501977-0674

(191) Sjobbema C, van der Mei S, Cornelius B, van der Klink J, Brouwer S. Exploring participatory behaviour of disability benefit claimants from an insurance physician's perspective. Disabil Rehabil (2018) 40:1943-1952. doi: 10.1080/09638288.2017.1323024

(192) Smith EM, Sakakibara BM, Miller WC. A review of factors influencing participation in social and community activities for wheelchair users. Disabil Rehabil Assist Technol (2016) 11:361-374. doi: 10.3109/17483107.2014.989420

(193) Soh SE, Barker AL, Morello RT, Ackerman IN. Applying the International Classification of Functioning, Disability and Health framework to determine the predictors of falls and fractures in people with osteoarthritis or at high risk of developing osteoarthritis: data from the Osteoarthritis Initiative. BMC Musculoskelet Disord (2020) 21:138-5. doi: 10.1186/s12891-020-3160-5

(194) Stamm TA, Mattsson M, Mihai C, Stöcker J, Binder A, Bauernfeind B, et al. Concepts of functioning and health important to people with systemic sclerosis: a qualitative study in four European countries. Ann Rheum Dis (2011) 70:1074-1079. doi: 10.1136/ard.2010.148767

(195) Stephens D, Pyykko I, Varpa K, Levo H, Poe D, Kentala E. Self-reported effects of Ménière's disease on the individual's life: a qualitative analysis. Otol Neurotol (2010) 31:335-338. doi: 10.1097/MAO.0b013e3181bc35ec

(196) Stergiou-Kita M, Grigorovich A, Gomez M. Development of an inter-professional clinical practice guideline for vocational evaluation following severe burn. Burns (2014) 40:1149-1163. doi: 10.1016/j.burns.2014.01.001

(197) Sumathipala K, Radcliffe E, Sadler E, Wolfe CD, McKevitt C. Identifying the long-term needs of stroke survivors using the International Classification of Functioning, Disability and Health. Chronic Illn (2012) 8:31-44. doi: 10.1177/1742395311423848

(198) Sung C, Chan F, Ditchman N, Chan C, Rumrill Jr PD. Evaluating the World Health Organization's International Classification of Functioning, Disability, and Health (ICF) framework as an employment model for people with epilepsy. J Vocat Rehabil (2020) 52:205-221. doi: 10.3233/JVR-201072

(199) Suttiwong J, Vongsirinavarat M, Hiengkaew V. Predictors of Community Participation Among Individuals With First Stroke: A Thailand Study. Ann Rehabil Med (2018) 42:660-669. doi: 10.5535/arm.2018.42.5.660

(200) Sánchez J, Rosenthal DA, Tansey TN, Frain MP, Bezyak JL. Predicting quality of life in adults with severe mental illness: Extending the International Classification of Functioning, Disability, and Health. Rehabil Psychol (2016) 61:19-31. doi: 10.1037/rep0000059

(201) Tederko P, Krasuski M, Ptyushkin P, Selb M, Pawlak K, Skrzypczyk R, et al. Need for a comprehensive epidemiologic study of spinal cord injury in Poland: findings from a systematic review. Spinal Cord (2013) 51:802-808. doi: 10.1038/sc.2013.105

(202) Theis KA, Murphy L, Hootman JM, Wilkie R. Social participation restriction among US adults with arthritis: a population-based study using the International Classification of Functioning, Disability and Health. Arthritis Care Res (2013) 65:1059-1069. doi: 10.1002/acr.21977

(203) Toovey R, Spittle AJ, Nicolaou A, McGinley JL, Harvey AR. Training Two-Wheel Bike Skills in Children with Cerebral Palsy: A Practice Survey of Therapists in Australia. Phys Occup Ther Pediatr (2019) 39:580-597. doi: 10.1080/01942638.2019.1585404

(204) Tschiesner U, Becker S, Cieza A. Health professional perspective on disability in head and neck cancer. Arch Otolaryngol Head Neck Surg (2010) 136:576-583. doi: 10.1001/archoto.2010.78

(205) Tseng MH, Chen KL, Shieh JY, Lu L, Huang CY. The determinants of daily function in children with cerebral palsy. Res Dev Disabil (2011) 32:235-245. doi: 10.1016/j.ridd.2010.09.024

(206) van Amelsvoort, L. G. P. M., de Brouwer, C. P. M., Heerkens YF, Widdershoven GAM, Kant I. Fostering functioning of workers: A new challenge for prevention in occupational health. Work (2017) 57:153-156. doi: 10.3233/WOR-172549

(207) van Dijk MJ, Smorenburg NT, Visser B, Nijhuis-van der Sanden, M. W., Heerkens YF. Description of movement quality in patients with low back pain: A qualitative study as a first step to a practical definition. Physiother Theory Pract (2017) 33:227-237. doi: 10.1080/09593985.2017.1282998

(208) van Gorp M, E Roebroeck M, van Eck M, M Voorman J, Twisk JWR, J Dallmeijer A, et al. Childhood factors predict participation of young adults with cerebral palsy in domestic life and interpersonal relationships: a prospective cohort study. Disabil Rehabil (2020) 42:3162-3171. doi: 10.1080/09638288.2019.1585971

(209) van Leeuwen LM, Merkus P, Pronk M, van der Torn M, Maré M, Goverts ST, et al. Overlap and Nonoverlap Between the ICF Core Sets for Hearing Loss and Otology and Audiology Intake Documentation. Ear Hear (2017) 38:103-116. doi: 10.1097/AUD.0000000000000358

(210) van Uem JM, Marinus J, Canning C, van Lummel R, Dodel R, Liepelt-Scarfone I, et al. Health-Related Quality of Life in patients with Parkinson's disease--A systematic review based on the ICF model. Neurosci Biobehav Rev (2016) 61:26-34. doi: 10.1016/j.neubiorev.2015.11.014

(211) van Wely L, van Gorp M, Tan SS, van Meeteren J, Roebroeck ME, Dallmeijer AJ. Teenage predictors of participation of adults with cerebral palsy in domestic life and interpersonal relationships: A 13-year follow-up study. Res Dev Disabil (2020) 96:103510. doi: 10.1016/j.ridd.2019.103510

(212) Vander Werff Kathy R. The Application of the International Classification of Functioning, Disability and Health to Functional Auditory Consequences of Mild Traumatic Brain Injury. Semin Hear (2016) 37:216-232. doi: 10.1055/s-0036-1584409

(213) Vargus-Adams JN, Majnemer A. International Classification of Functioning, Disability and Health (ICF) as a framework for change: revolutionizing rehabilitation. J Child Neurol (2014) 29:1030-1035. doi: 10.1177/0883073814533595

(214) Verboom CE, Sentse M, Sijtsema JJ, Nolen WA, Ormel J, Penninx BW. Explaining heterogeneity in disability with major depressive disorder: effects of personal and environmental characteristics. J Affect Disord (2011) 132:71-81. doi: 10.1016/j.jad.2011.01.016

(215) Verma CV, Vora T, Thatte M, Yardi S. Patient perception after traumatic brachial plexus injury: A qualitative case study. J Hand Ther (2020) 33:593-597. doi: 10.1016/j.jht.2019.03.00

(216) Vieira ER, Berean C, Paches D, Costa L, Décombas-Deschamps N, Caveny P, et al. Risks and suggestions to prevent falls in geriatric rehabilitation: a participatory approach. BMJ Qual Saf (2011) 20:440-448. doi: 10.1136/bmjqs.2010.042382

(217) Virués-Ortega J, de Pedro-Cuesta J, del Barrio JL, Almazan-Isla J, Bergareche A, Bermejo-Pareja F, et al. Medical, environmental and personal factors of disability in the elderly in Spain: a screening survey based on the International Classification of Functioning. Gac Sanit (2011) 25 Suppl 2:29-38. doi: 10.1016/j.gaceta.2011.07.021

(218) Vooijs M, Leensen MC, Hoving JL, Daams JG, Wind H, Frings-Dresen MH. Disease-generic factors of work participation of workers with a chronic disease: a systematic review. Int Arch Occup Environ Health (2015) 88:1015-1029. doi: 10.1007/s00420-015-1025-2

(219) Wahlgren A, Palombaro K. Evidence-based physical therapy for BPPV using the International Classification of Functioning, Disability and Health model: a case report. J Geriatr Phys Ther (2012) 35:200-205. doi: 10.1519/JPT.0b013e318247a243

(220) Wang Y-T, Lin Y-J. Employment Outcome Predictors for People with Disabilities in Taiwan - A Preliminary Study Using ICF Conceptual Frameworks. J Rehabil (2013) 79:3-14.

(221) Wang YC, Kapellusch J, Garg A. Important factors influencing the return to work after stroke. Work (2014) 47:553-559. doi: 10.3233/WOR-131627

(222) Warnink-Kavelaars J, Beelen A, Dekker S, Nollet F, Menke LA, Engelbert RHH. Marfan syndrome in childhood: parents' perspectives of the impact on daily functioning of children, parents and family; a qualitative study. BMC Pediatr (2019) 19:262-6. doi: 10.1186/s12887-019-1612-6

(223) Willis J, Hophing L, Mahlberg N, Ronen GM. Youth with epilepsy: Their insight into participating in enhanced physical activity study. Epilepsy Behav (2018) 89:63-69. doi: 10.1016/j.yebeh.2018.10.011

(224) Worrall LE, Hudson K, Khan A, Ryan B, Simmons-Mackie N. Determinants of Living Well With Aphasia in the First Year Poststroke: A Prospective Cohort Study. Arch Phys Med Rehabil (2017) 98:235-240. doi: 10.1016/j.apmr.2016.06.020

(225) Yen TH, Lin LF, Wei TS, Chang KH, Wang YH, Liou TH. Delphi-based assessment of fall-related risk factors in acute rehabilitation settings according to the International Classification of Functioning, Disability and Health. Arch Phys Med Rehabil (2014) 95:50-57. doi: 10.1016/j.apmr.2013.09.006

(226) Zhu W, Jiang Y. Determinants of quality of life in patients with hemorrhagic stroke: A path analysis. Medicine (Baltimore) (2019) 98:e13928. doi: 10.1097/MD.0000000000013928
